# Supplementary figures and images for: Increasing Cellular Uptake and Permeation of Curcumin Using a Novel Polymer-Surfactant Formulation
Source: Biomolecules. 2022 Nov 23;12(12):1739. doi: 10.3390/biom12121739 (PMC9775279; doi:10.3390/biom12121739)

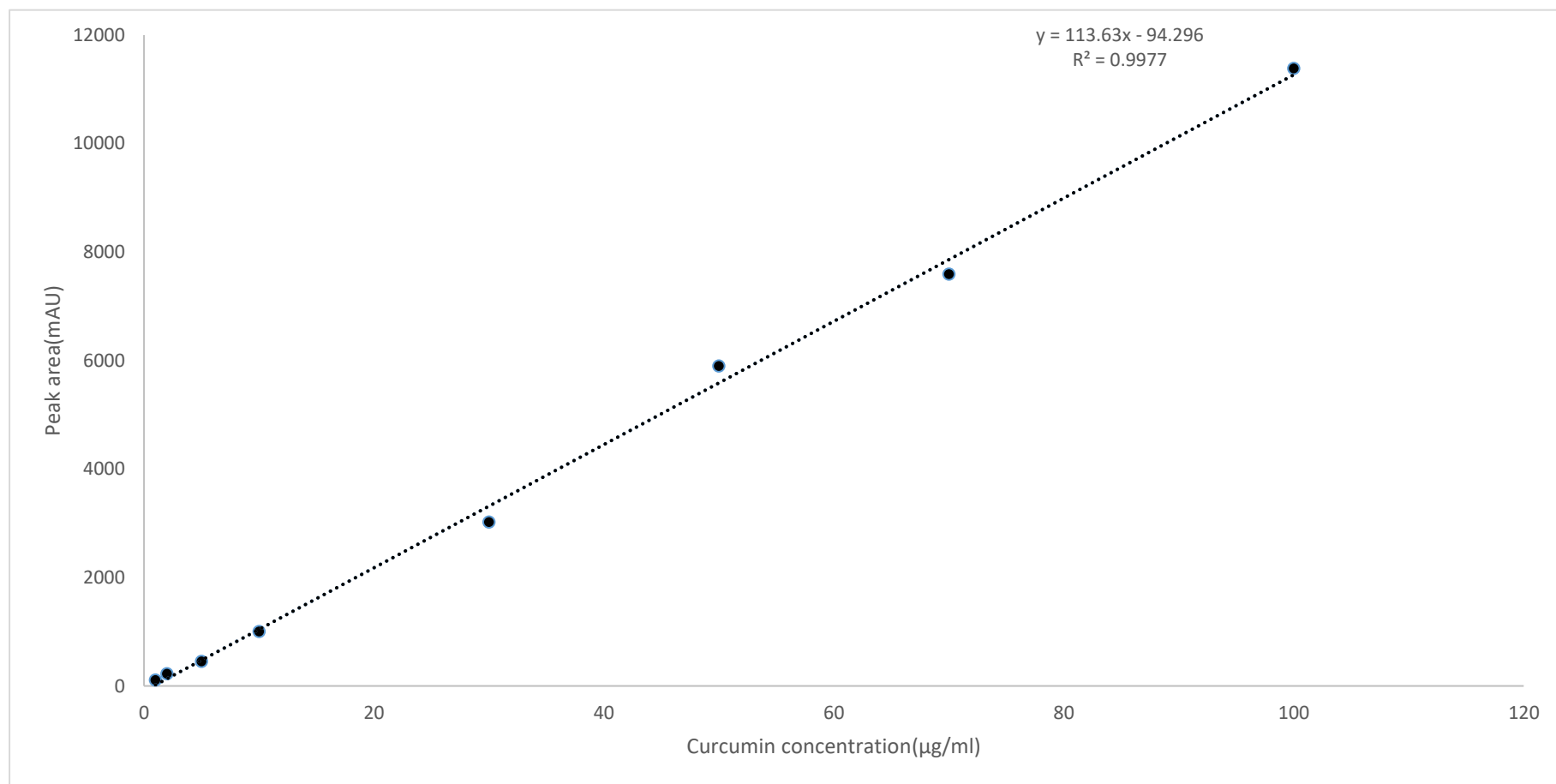

Supplement: Supplementary file 1 [file biomolecules-12-01739-s001.zip › Figure S1.pdf]

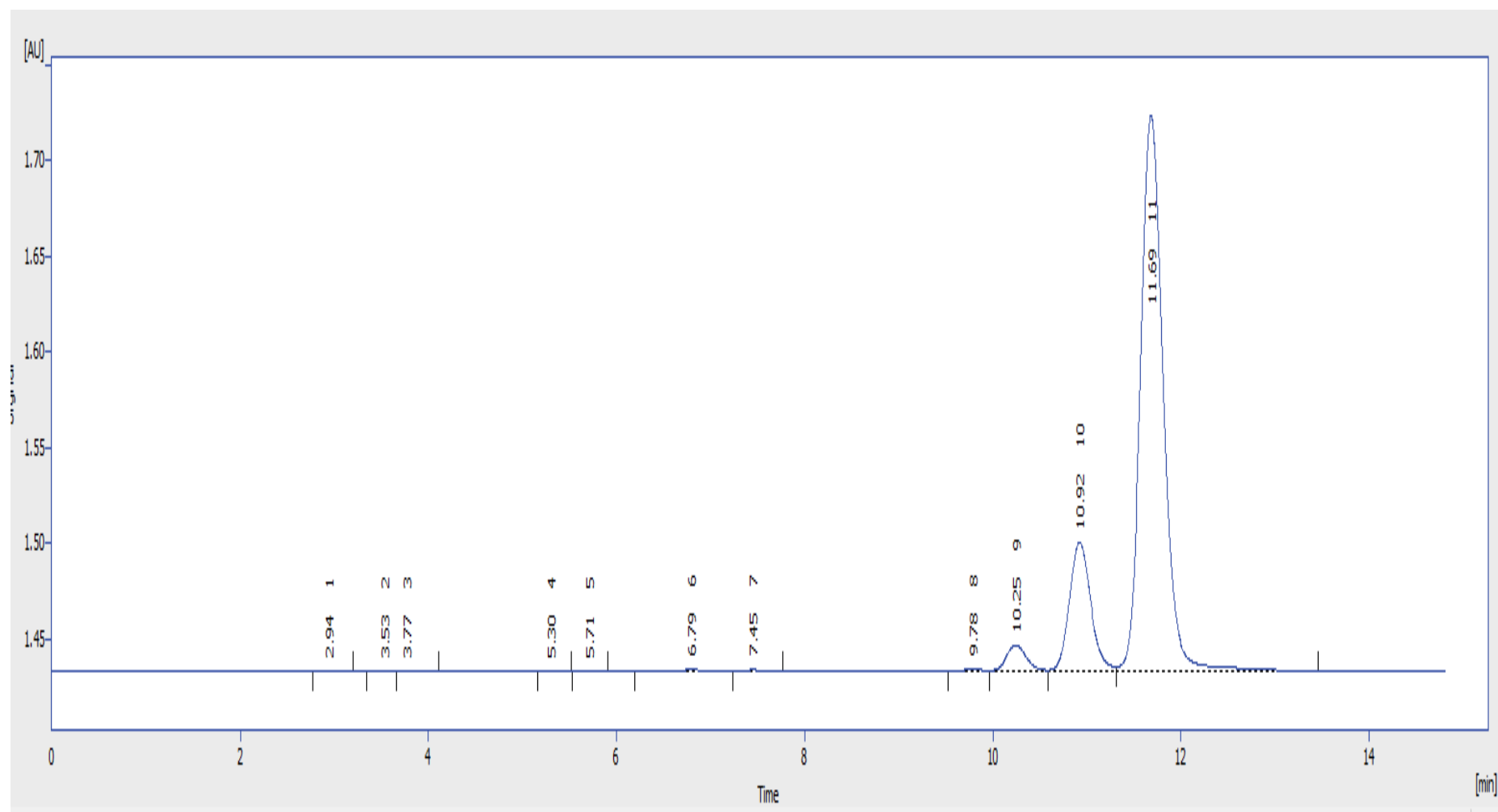

Supplement: Supplementary file 1 [file biomolecules-12-01739-s001.zip › Figure S2.pdf]

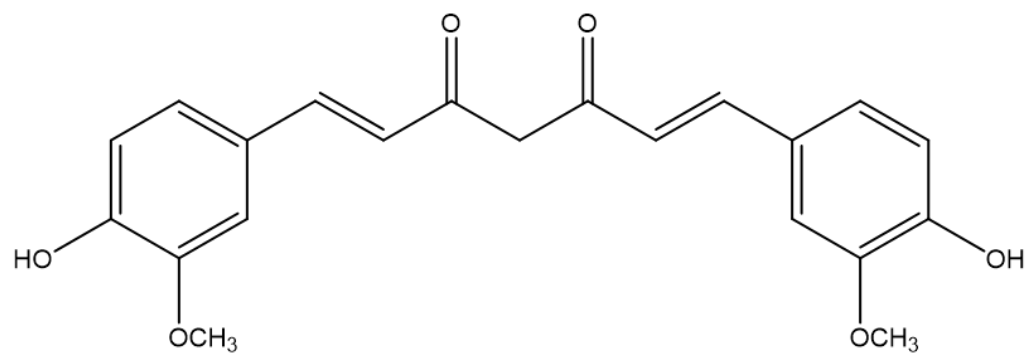

Keto form of Curcumin

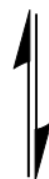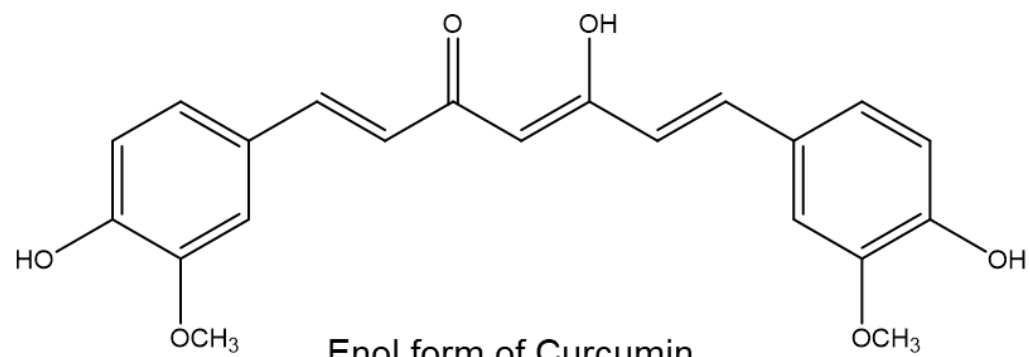

Enol form of Curcumin

Supplement: Supplementary file 1 [file biomolecules-12-01739-s001.zip › New Figure 1.pdf]

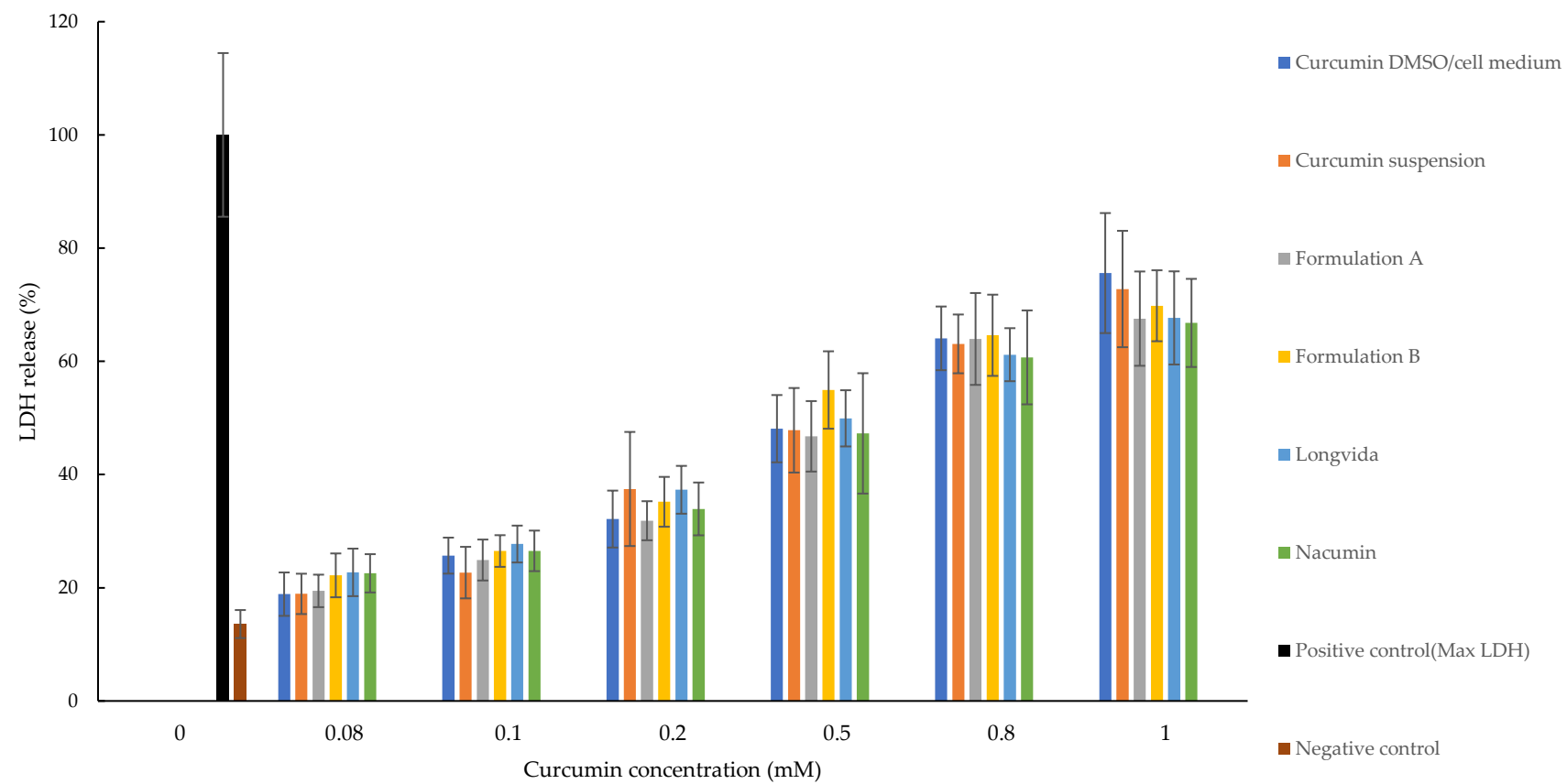

Supplement: Supplementary file 1 [file biomolecules-12-01739-s001.zip › New Figure 10.pdf]

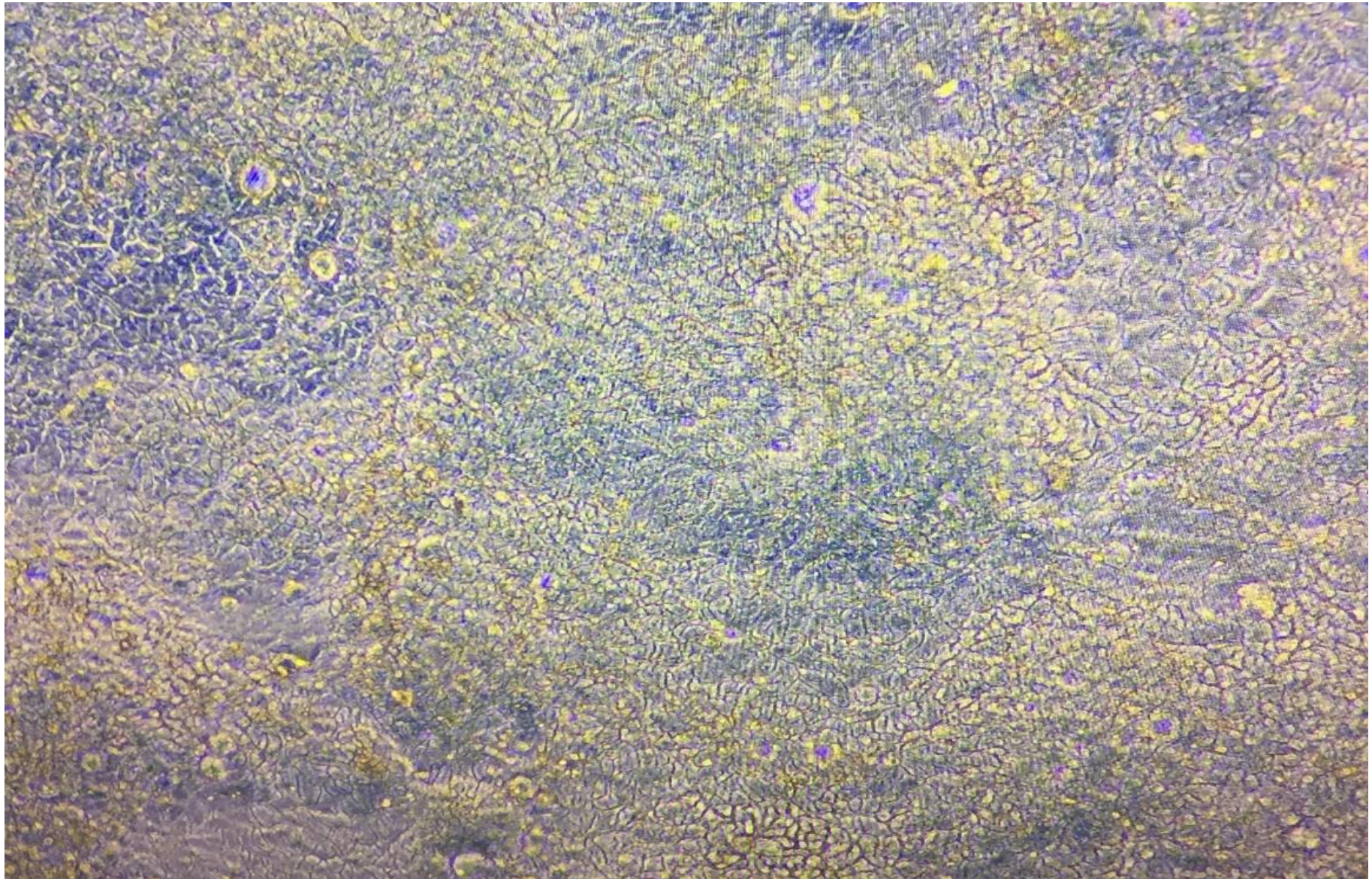

Supplement: Supplementary file 1 [file biomolecules-12-01739-s001.zip › New Figure 11.pdf]

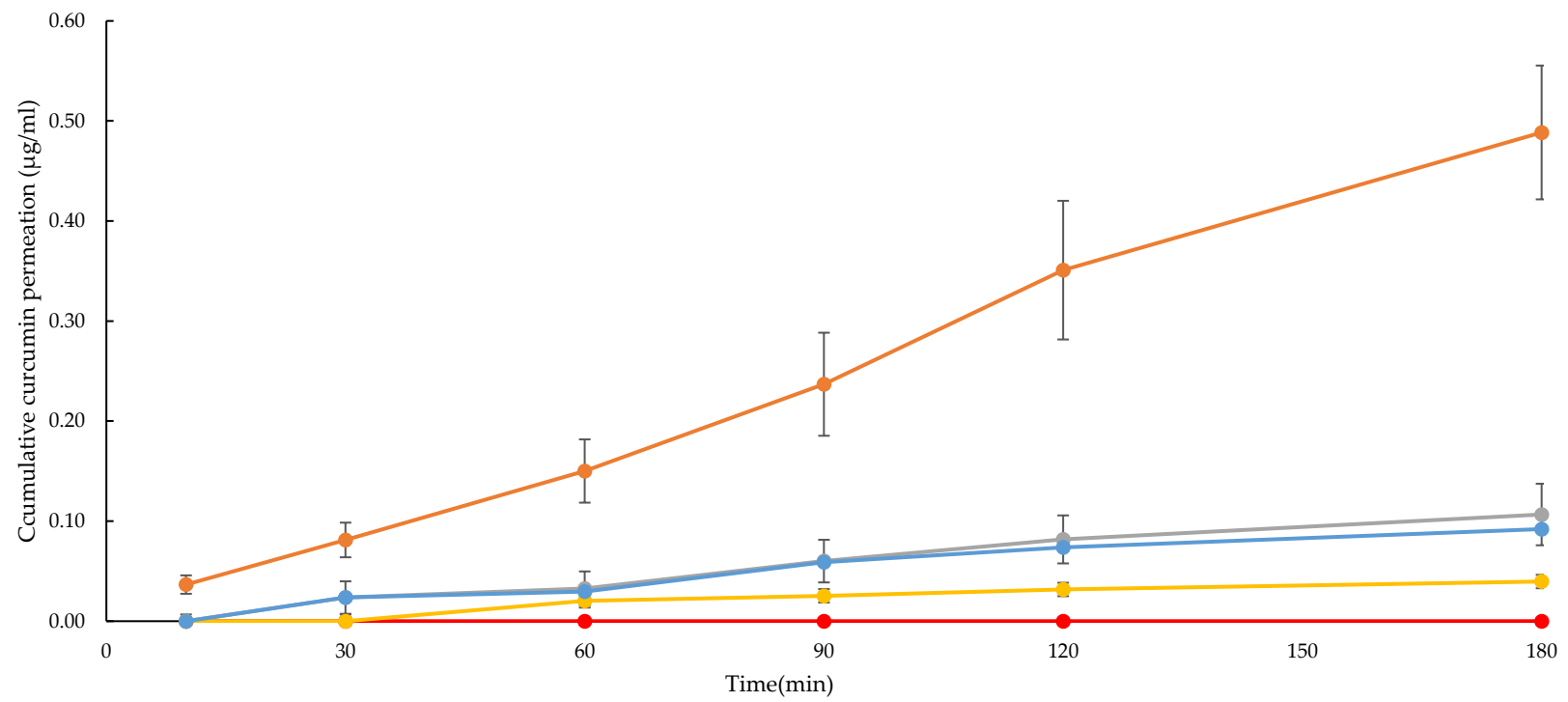

Commercial curcumin      Formulation A      Formulation B      Longvida      Nacumin

Supplement: Supplementary file 1 [file biomolecules-12-01739-s001.zip › New Figure 12.pdf]

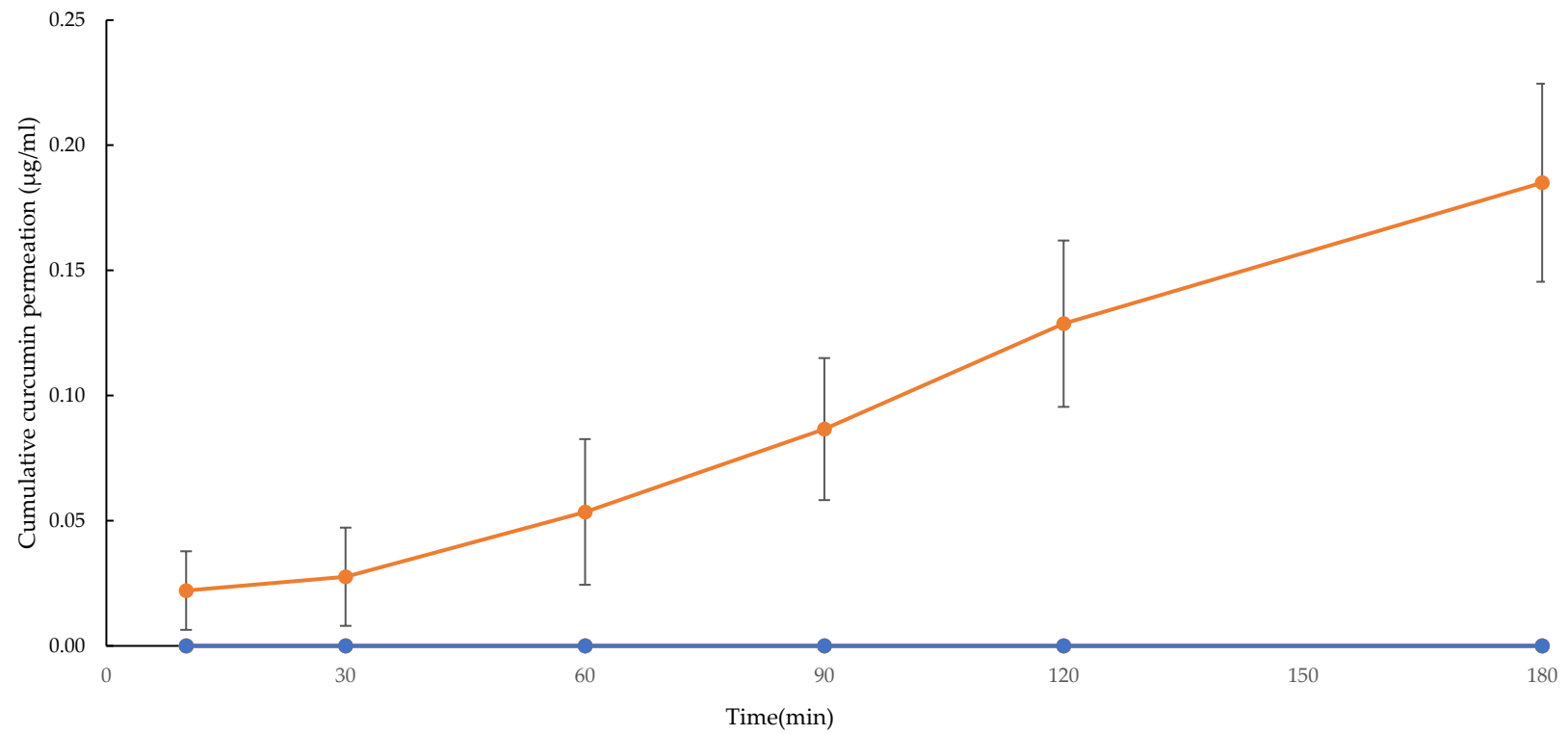

Commercial curcumin

Formulation A

Formulation B

Longvida

Nacumin

Supplement: Supplementary file 1 [file biomolecules-12-01739-s001.zip › New Figure 13.pdf]

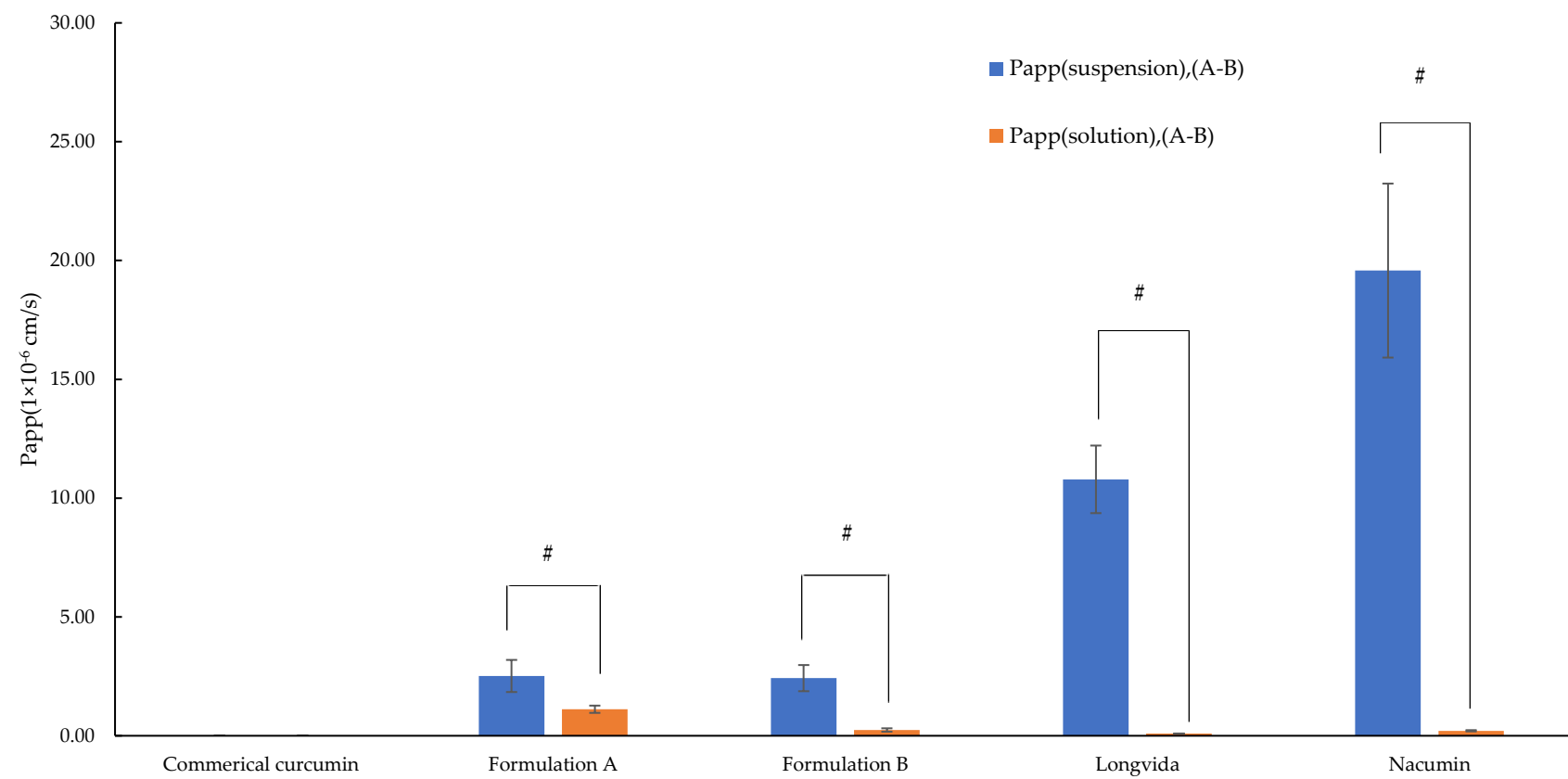

Supplement: Supplementary file 1 [file biomolecules-12-01739-s001.zip › New Figure 14.pdf]

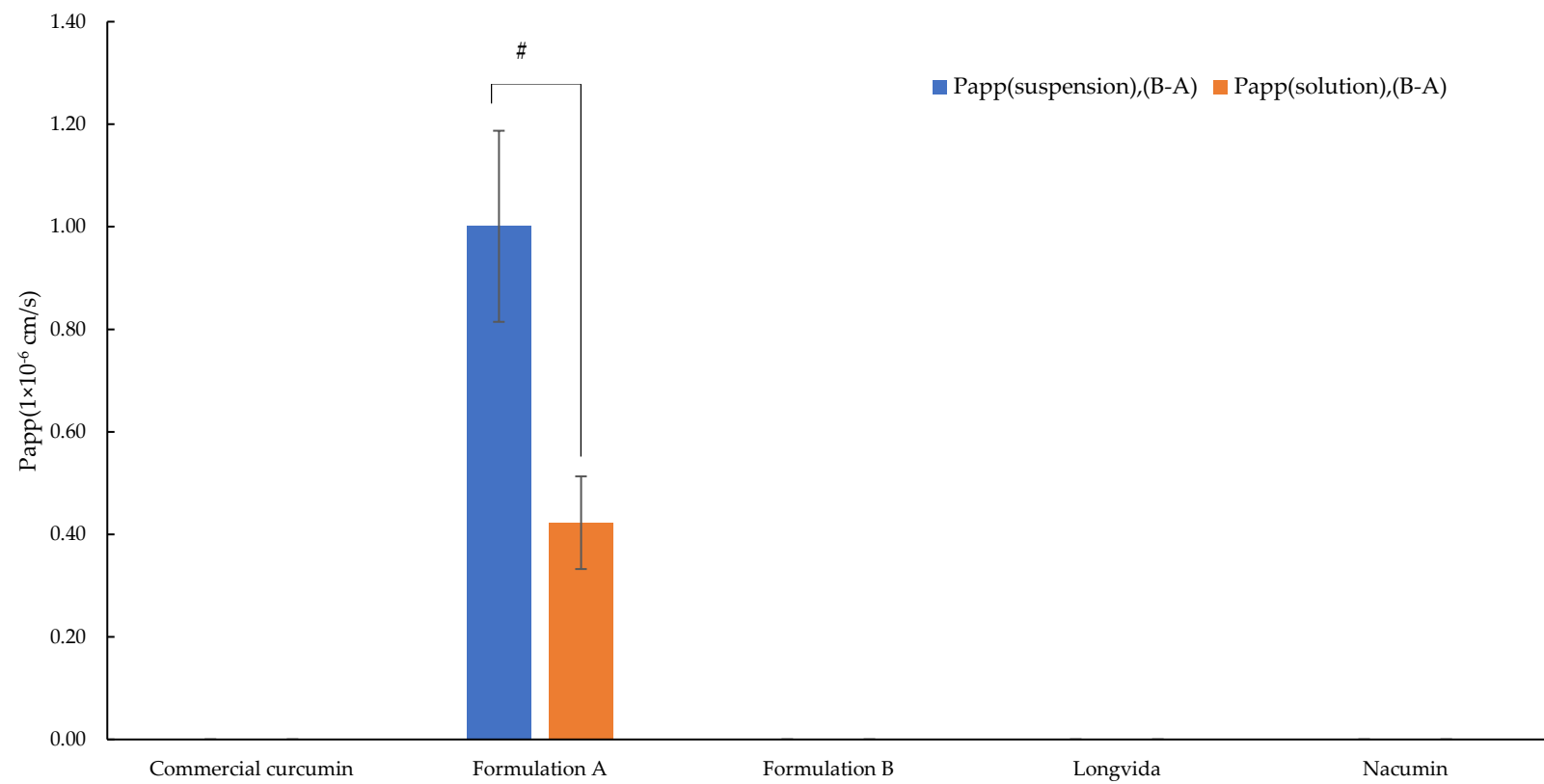

Supplement: Supplementary file 1 [file biomolecules-12-01739-s001.zip › New Figure 15.pdf]

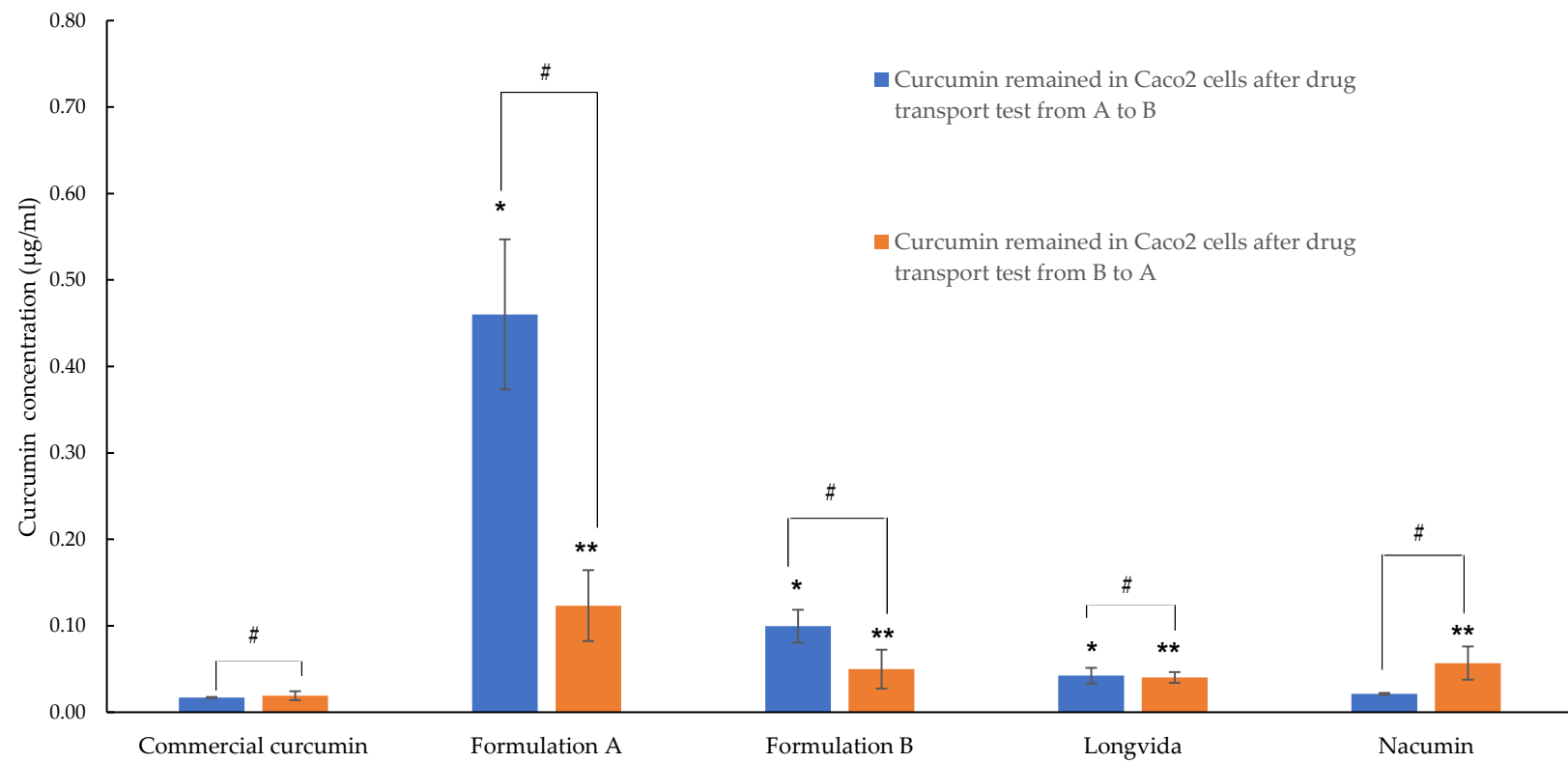

Supplement: Supplementary file 1 [file biomolecules-12-01739-s001.zip › New Figure 16.pdf]

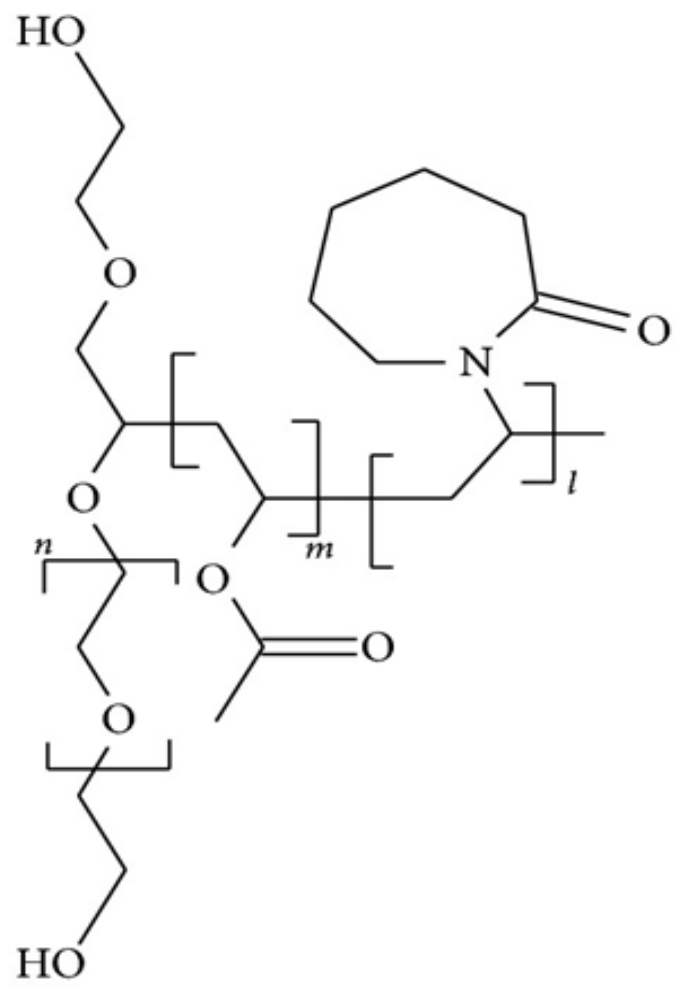

Supplement: Supplementary file 1 [file biomolecules-12-01739-s001.zip › New Figure 2.pdf]

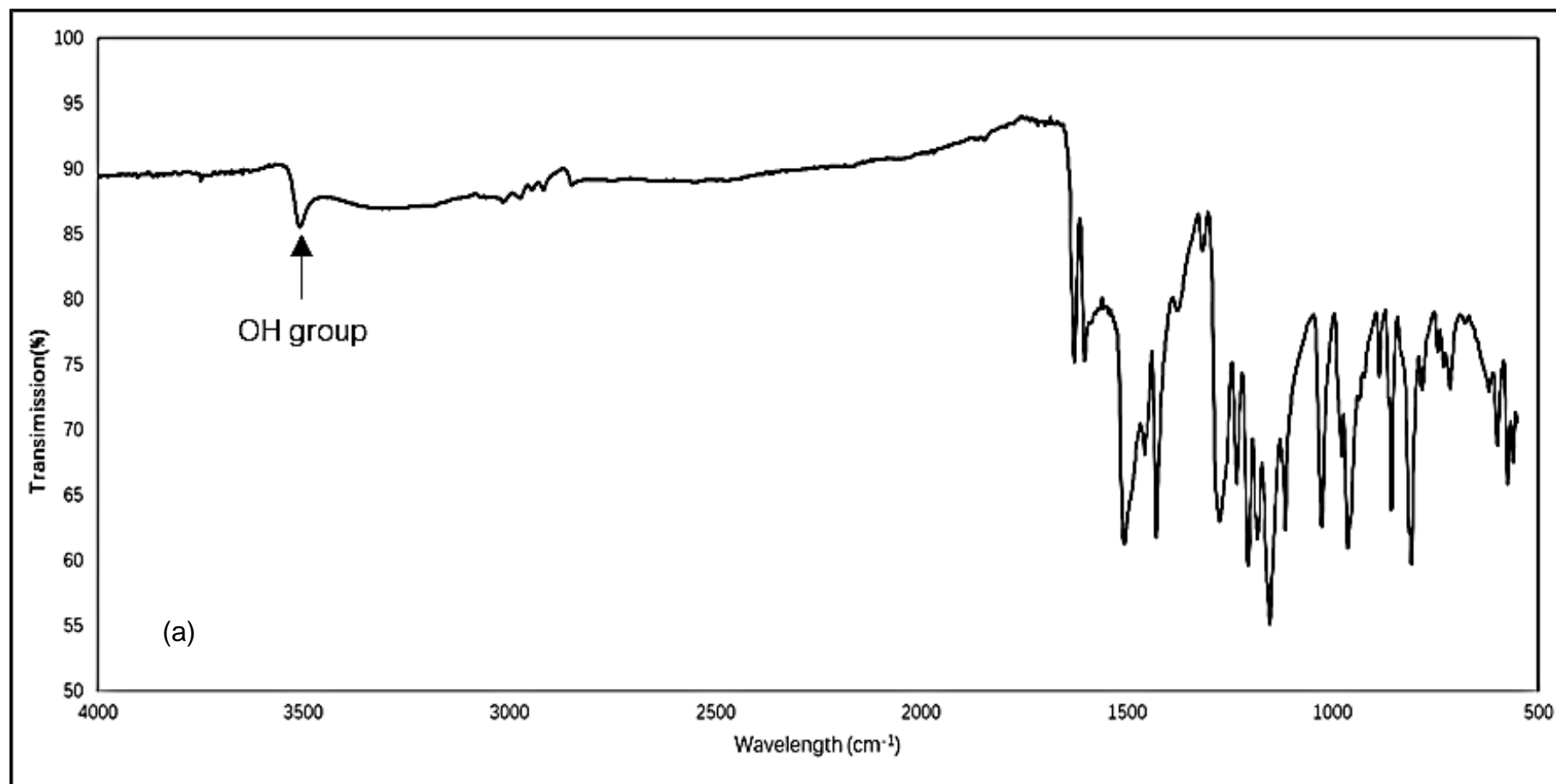

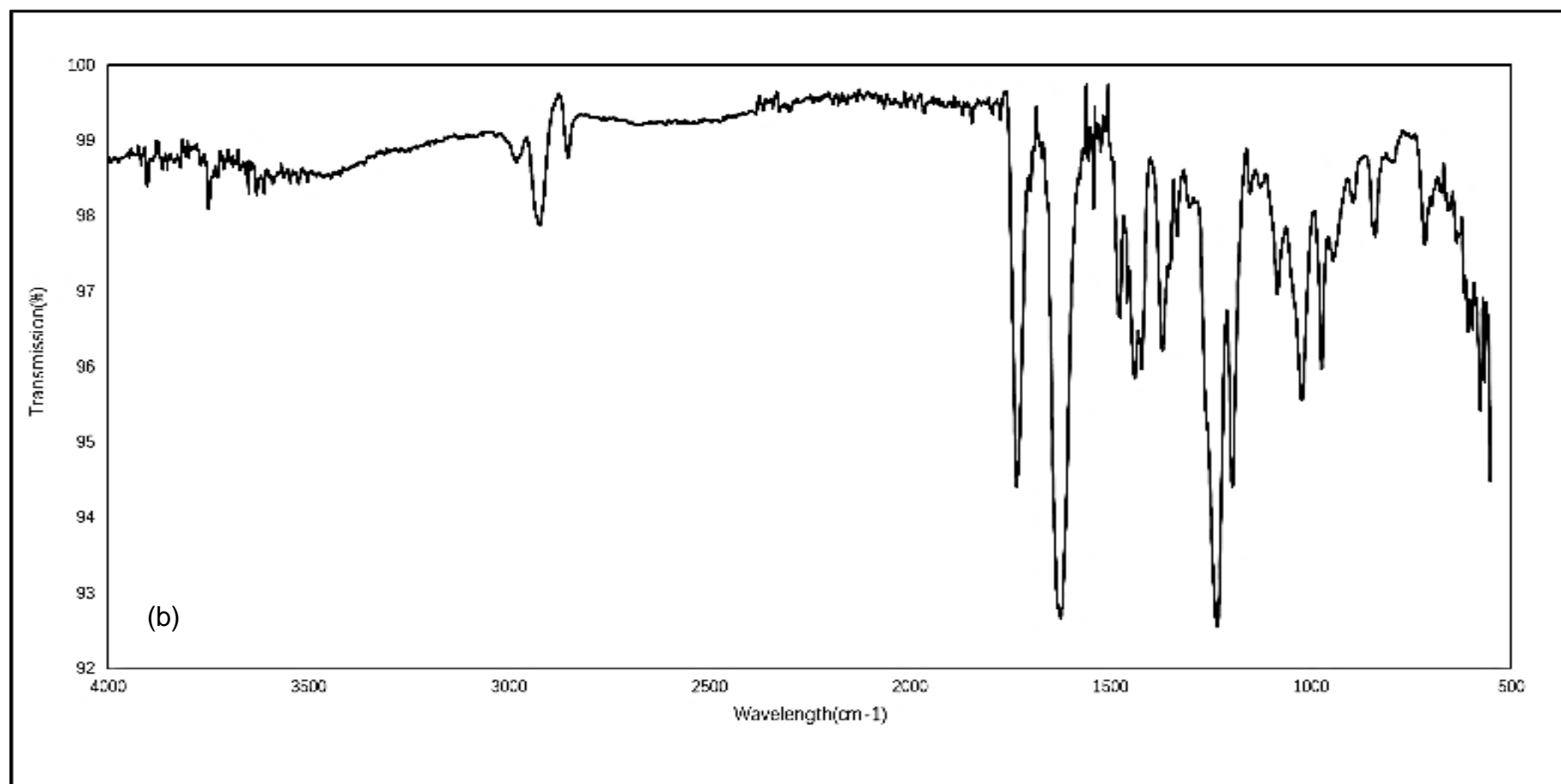

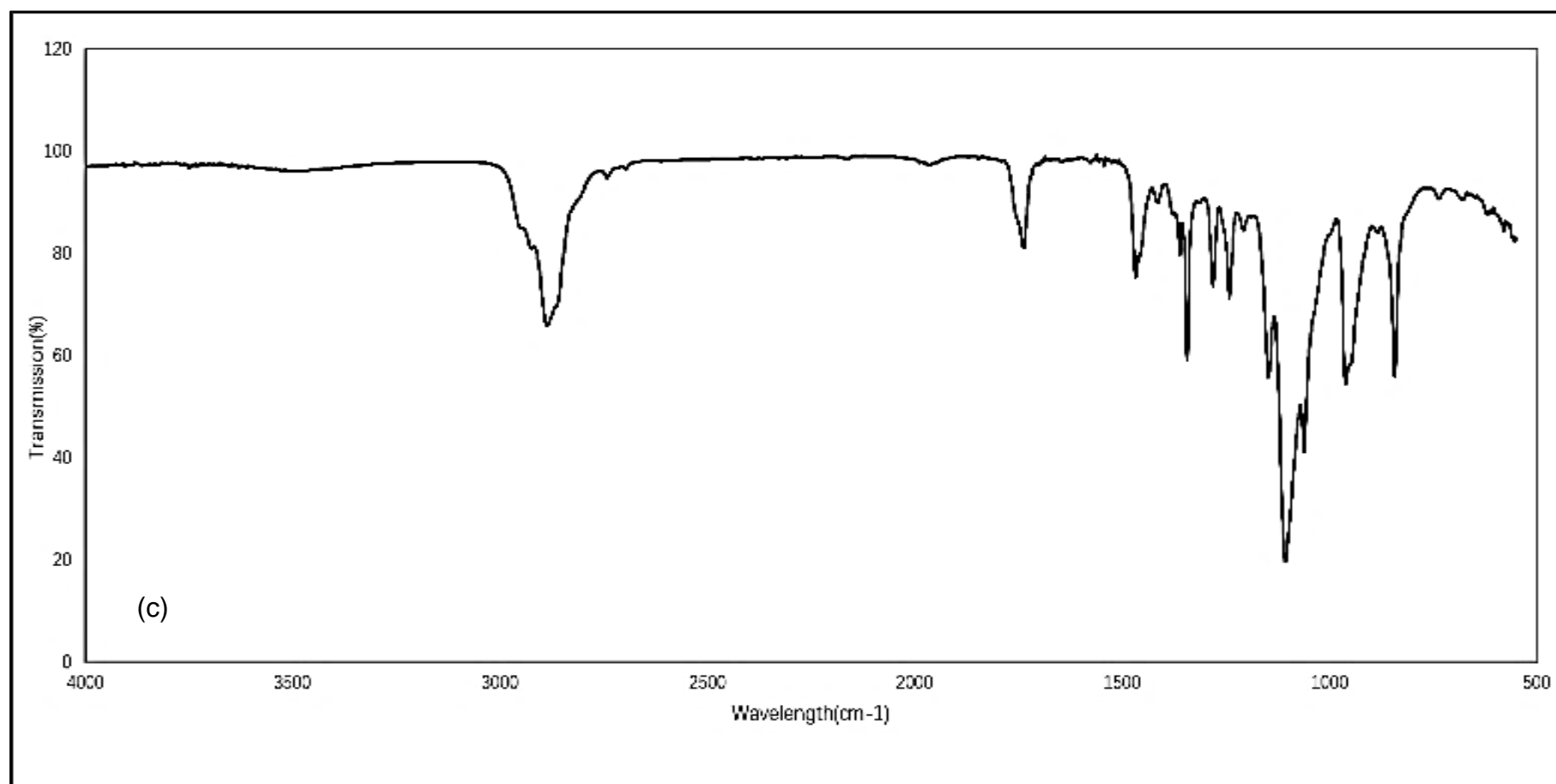

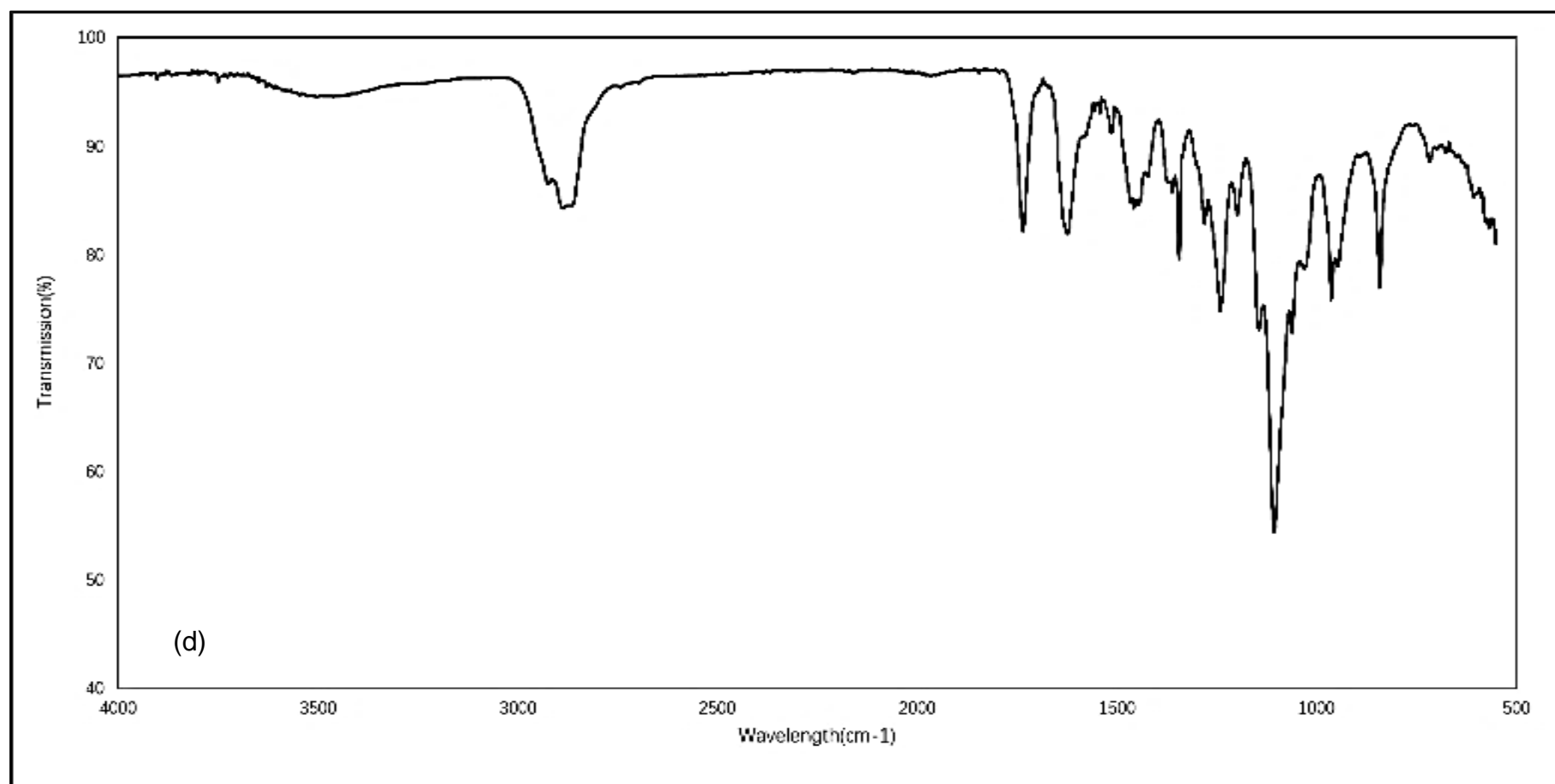

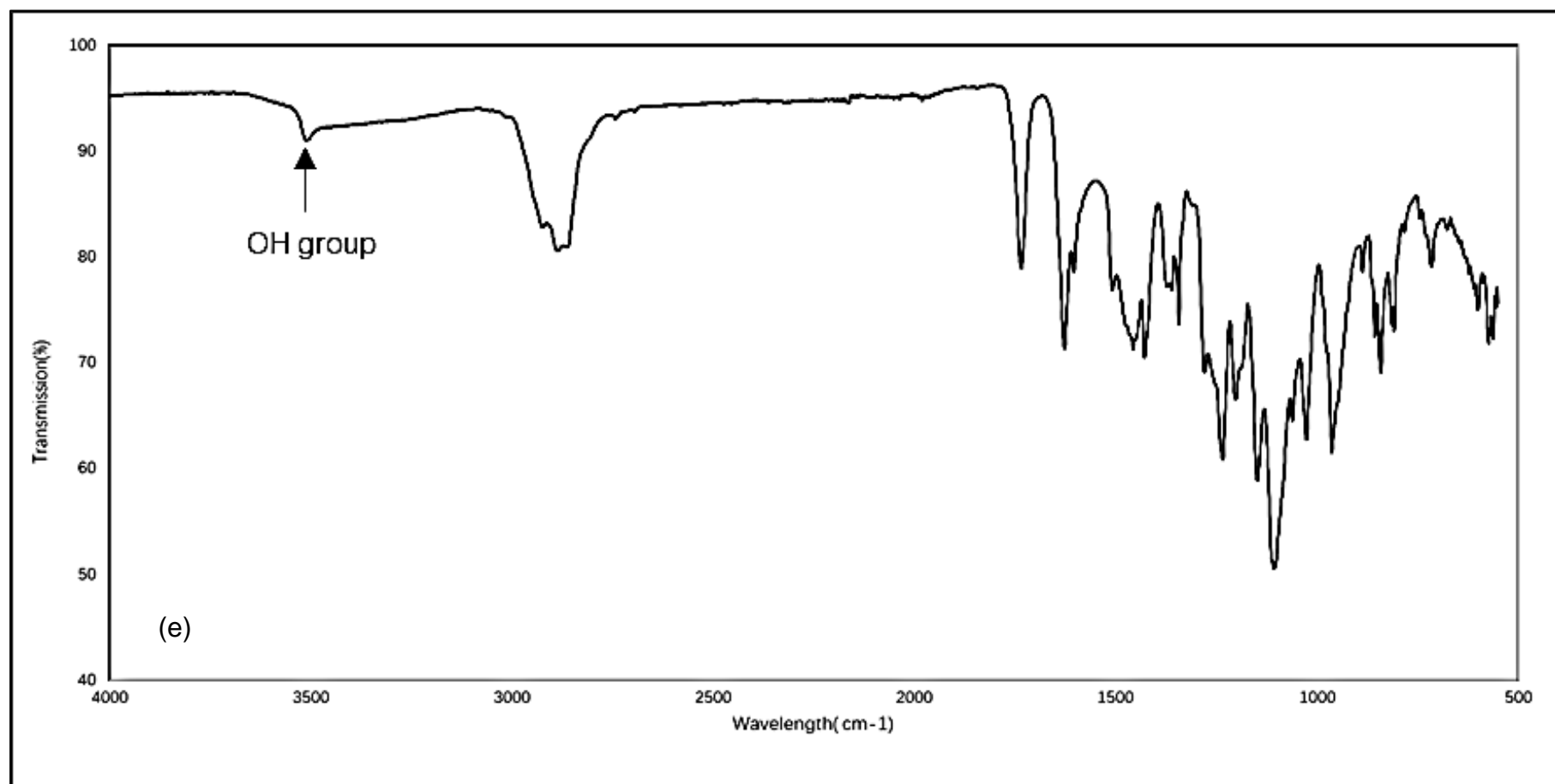

Supplement: Supplementary file 1 [file biomolecules-12-01739-s001.zip › New Figure 3.pdf]

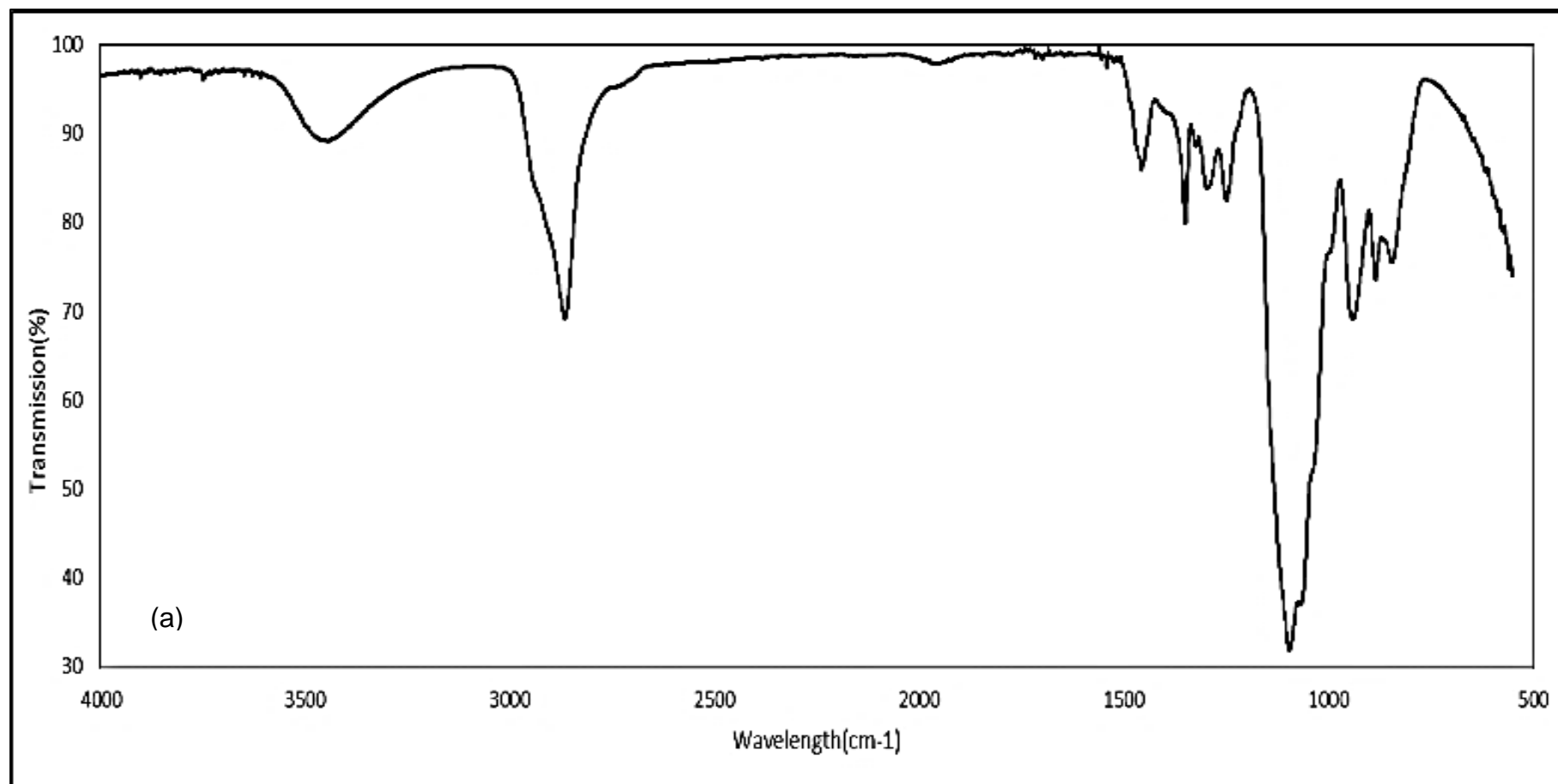

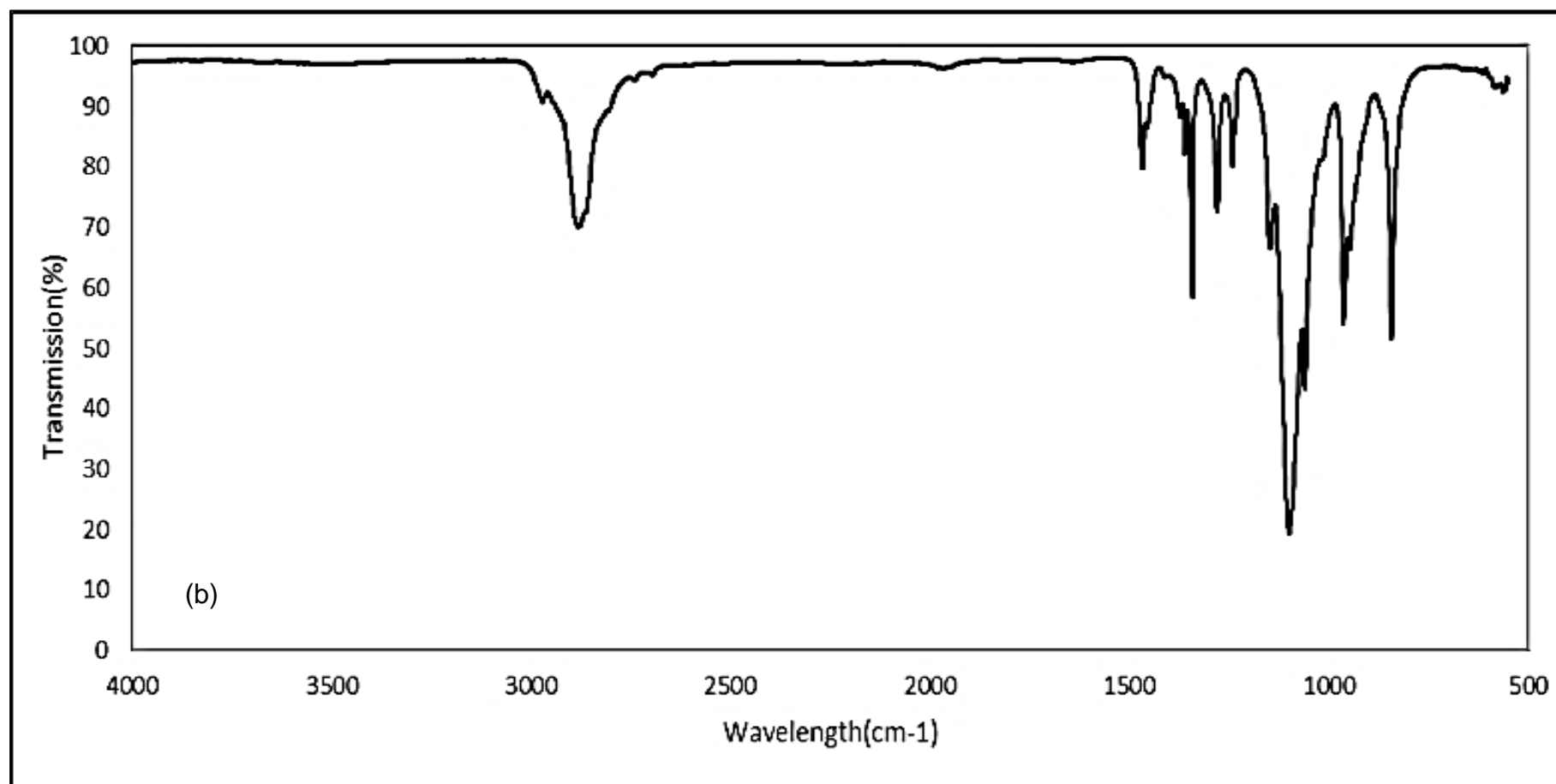

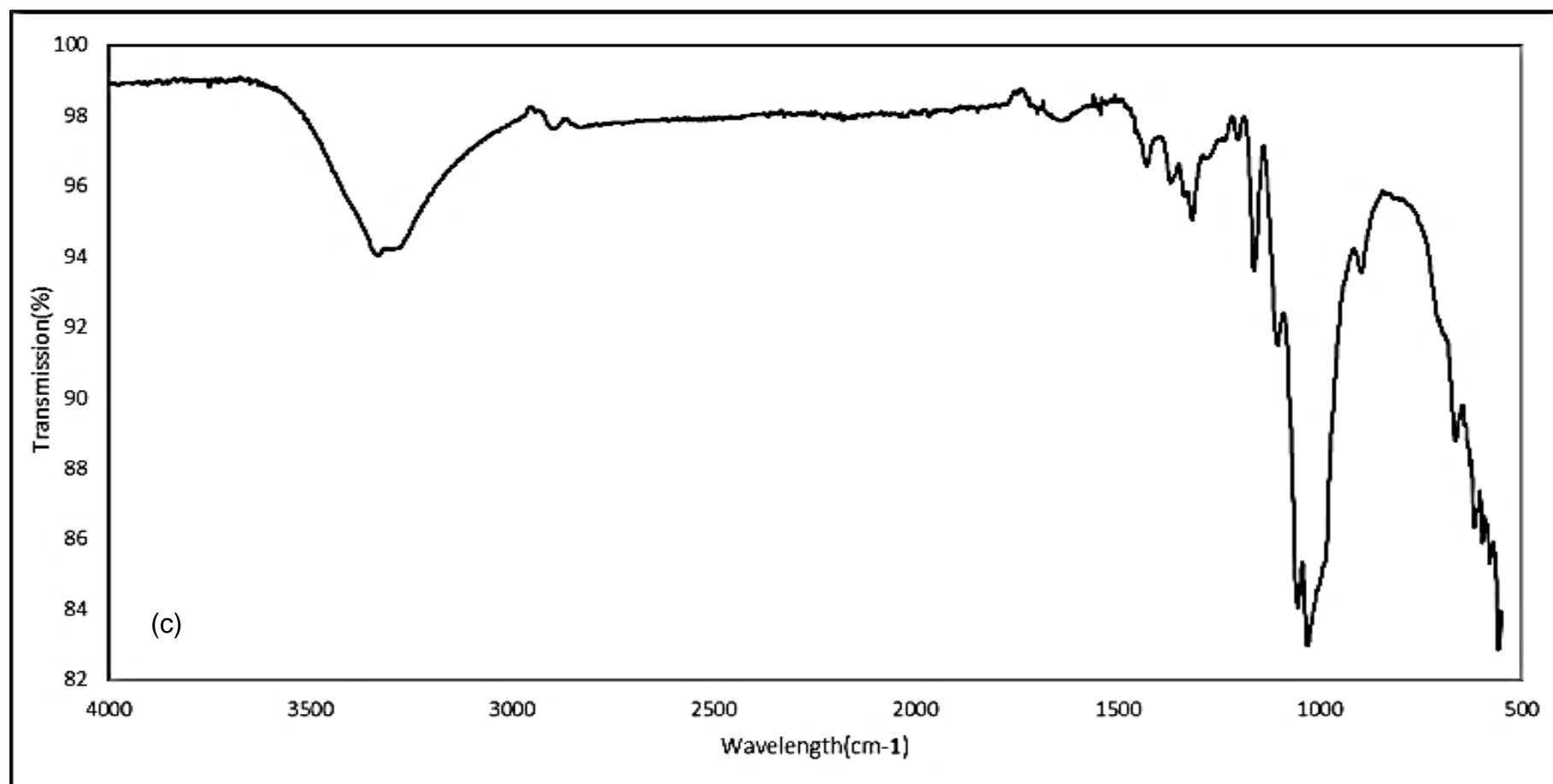

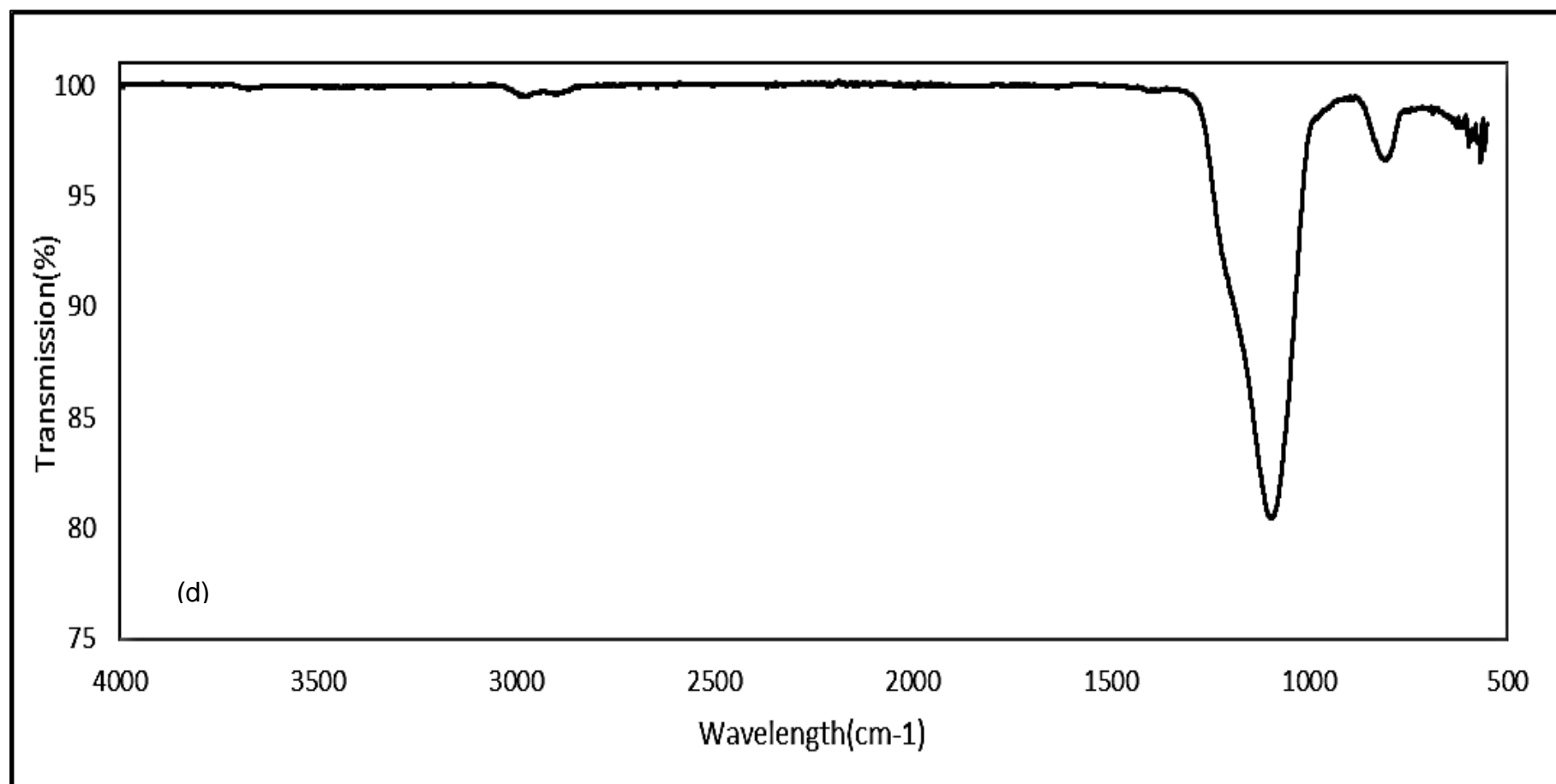

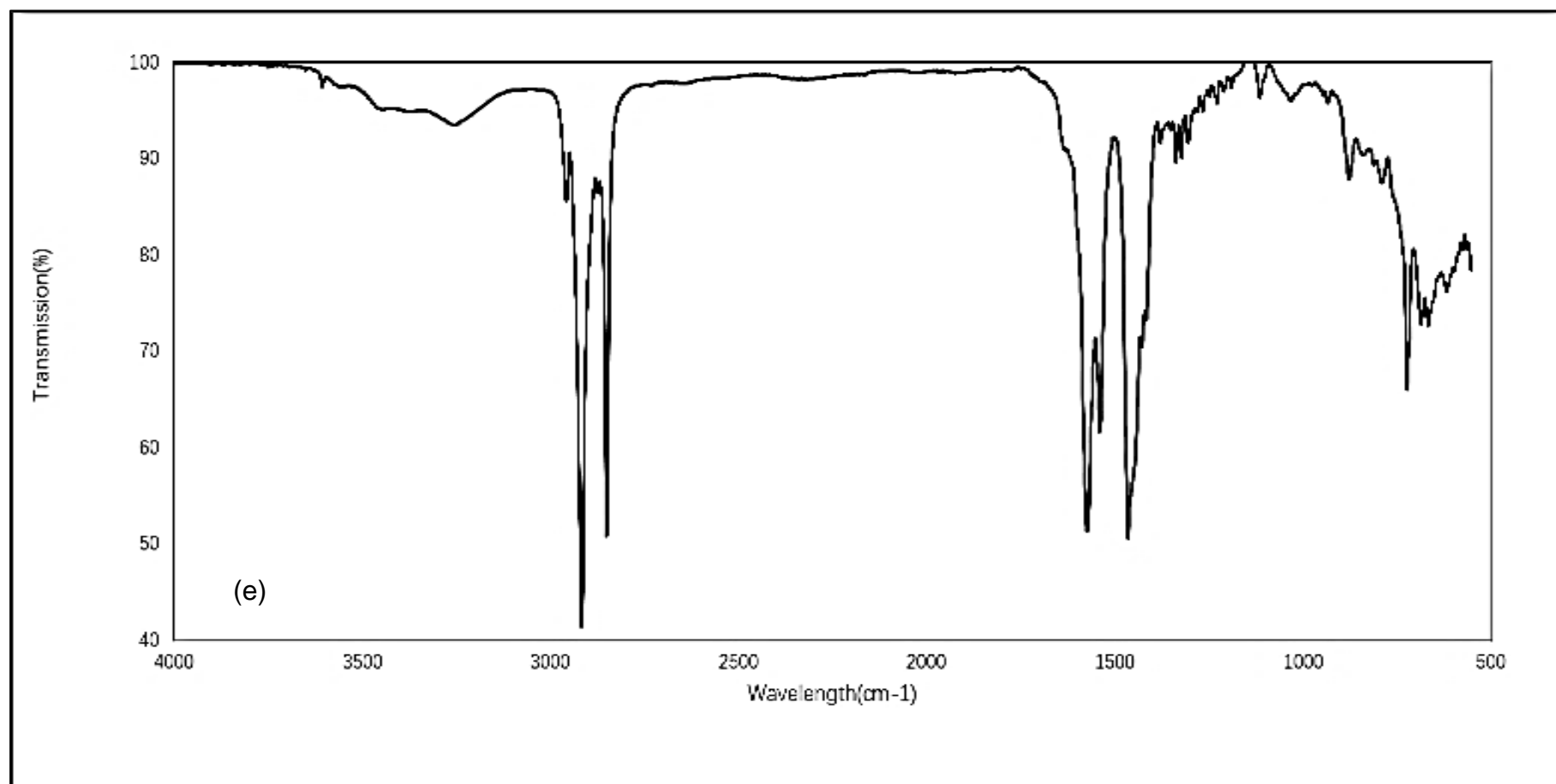

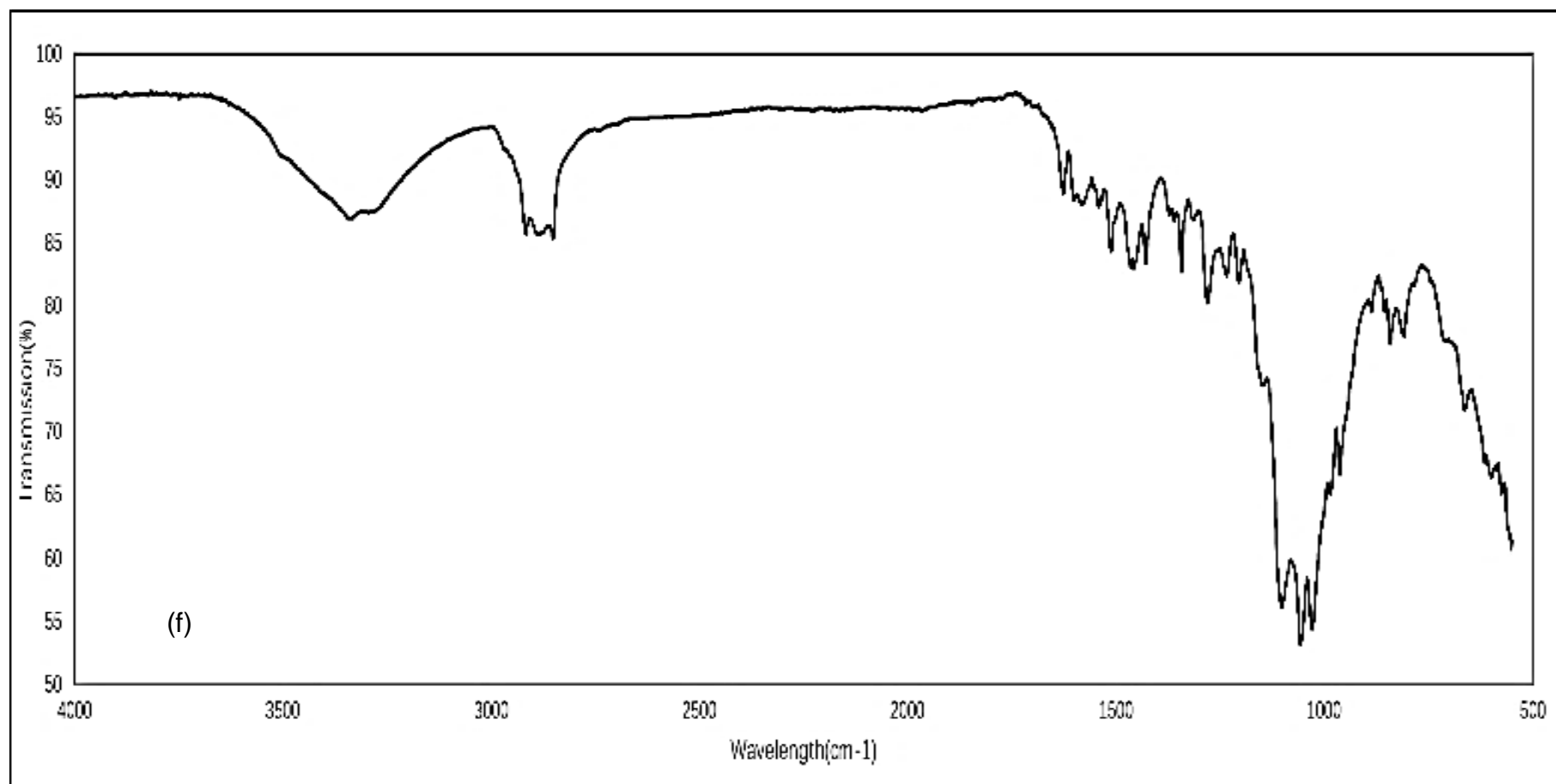

Supplement: Supplementary file 1 [file biomolecules-12-01739-s001.zip › New Figure 4.pdf]

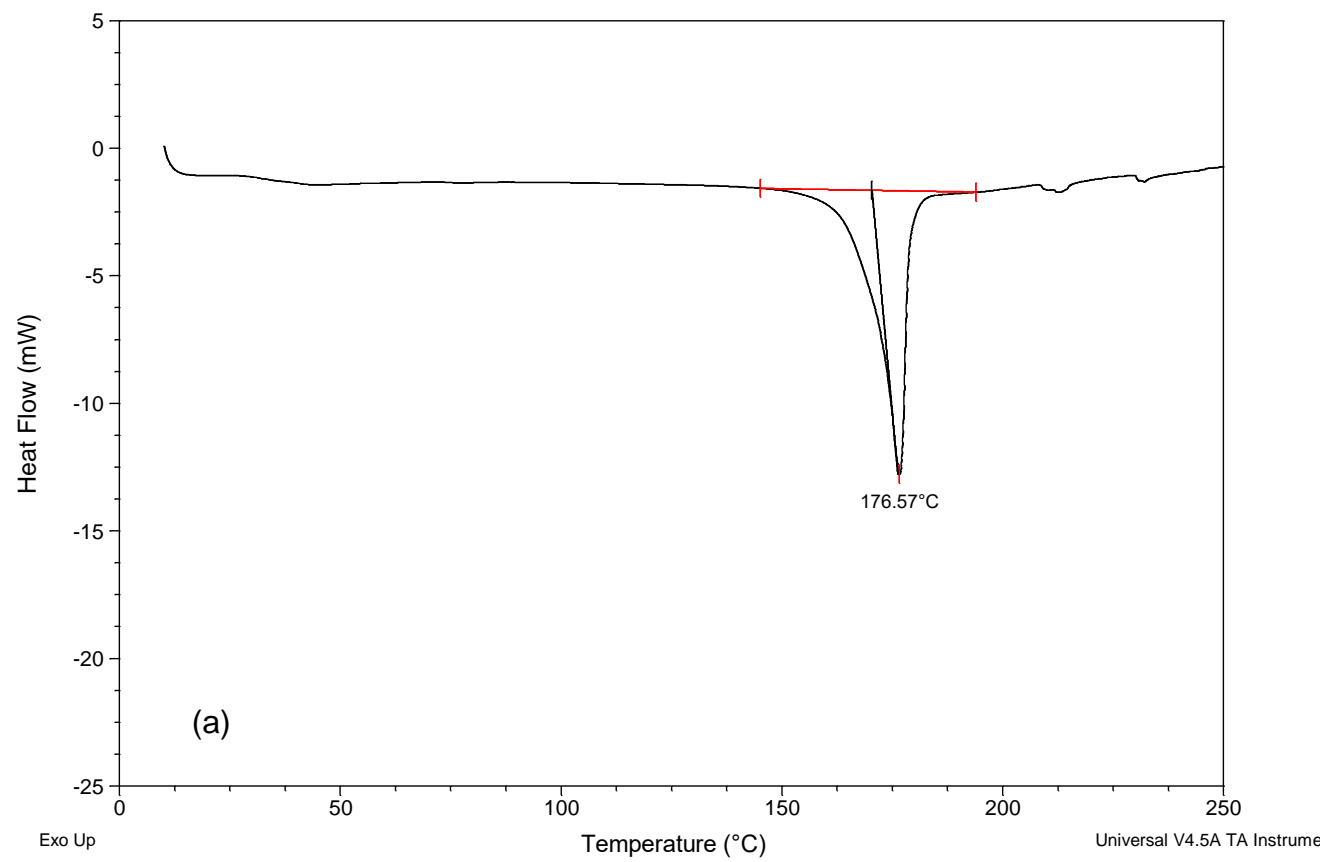

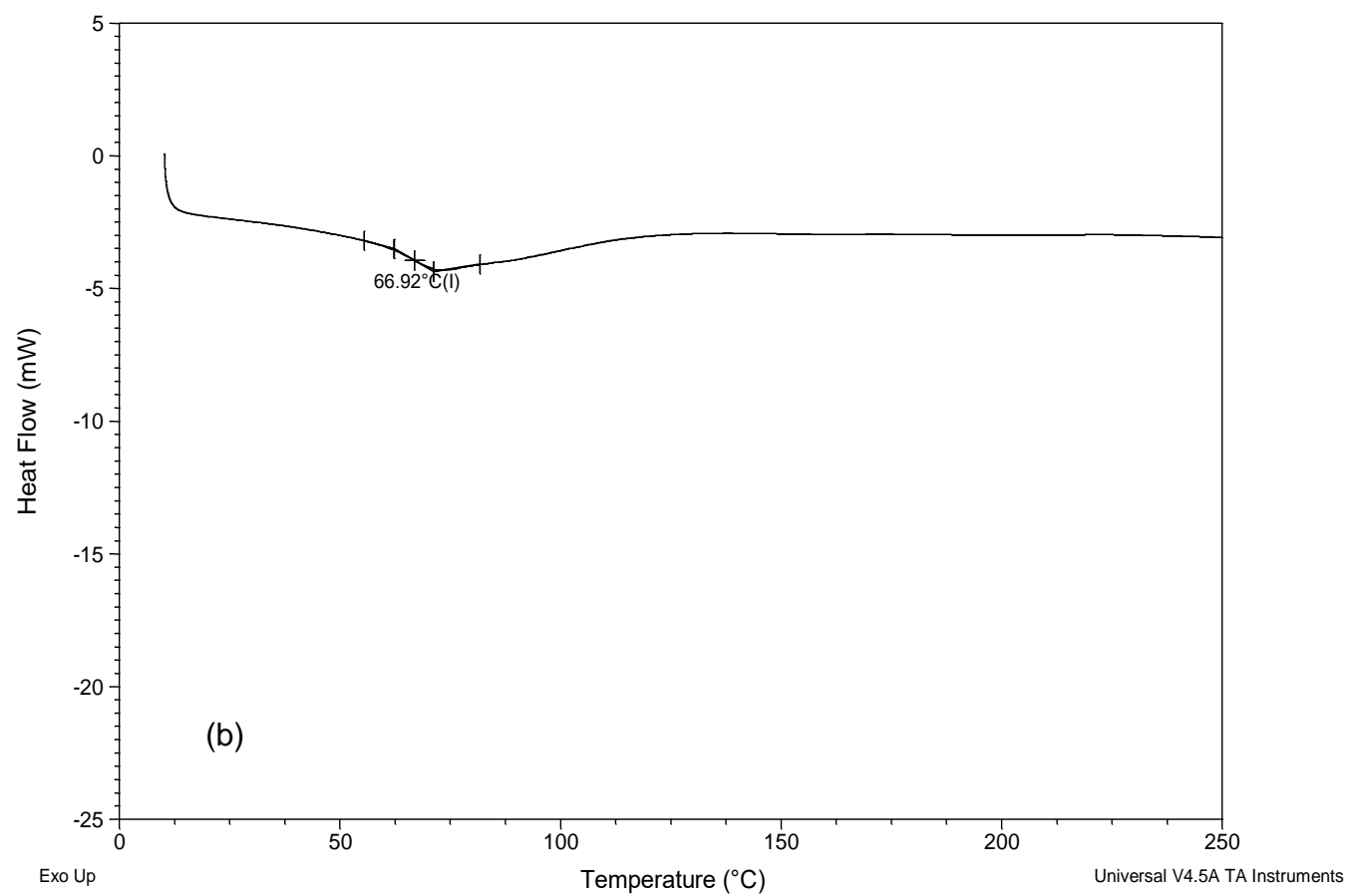

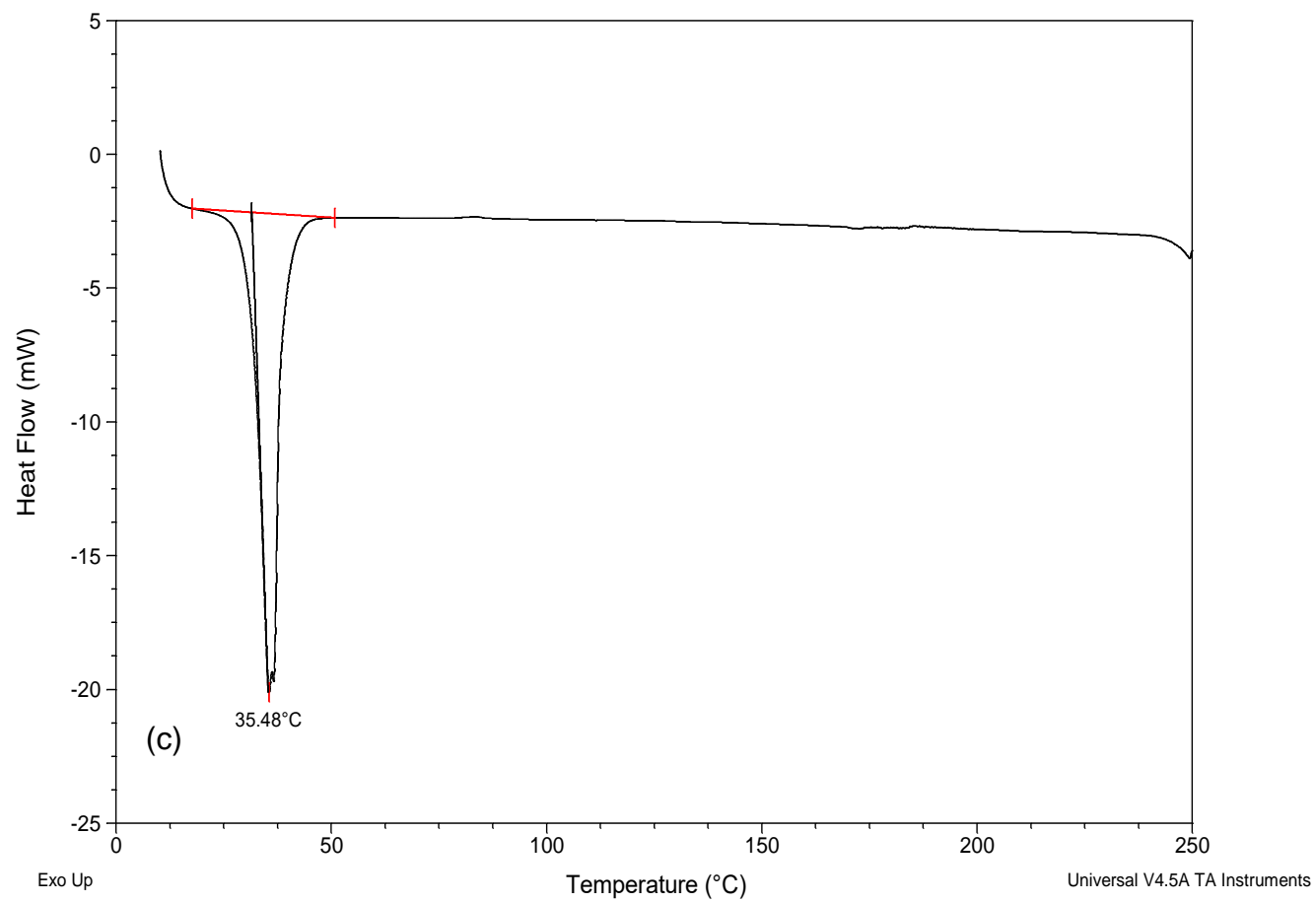

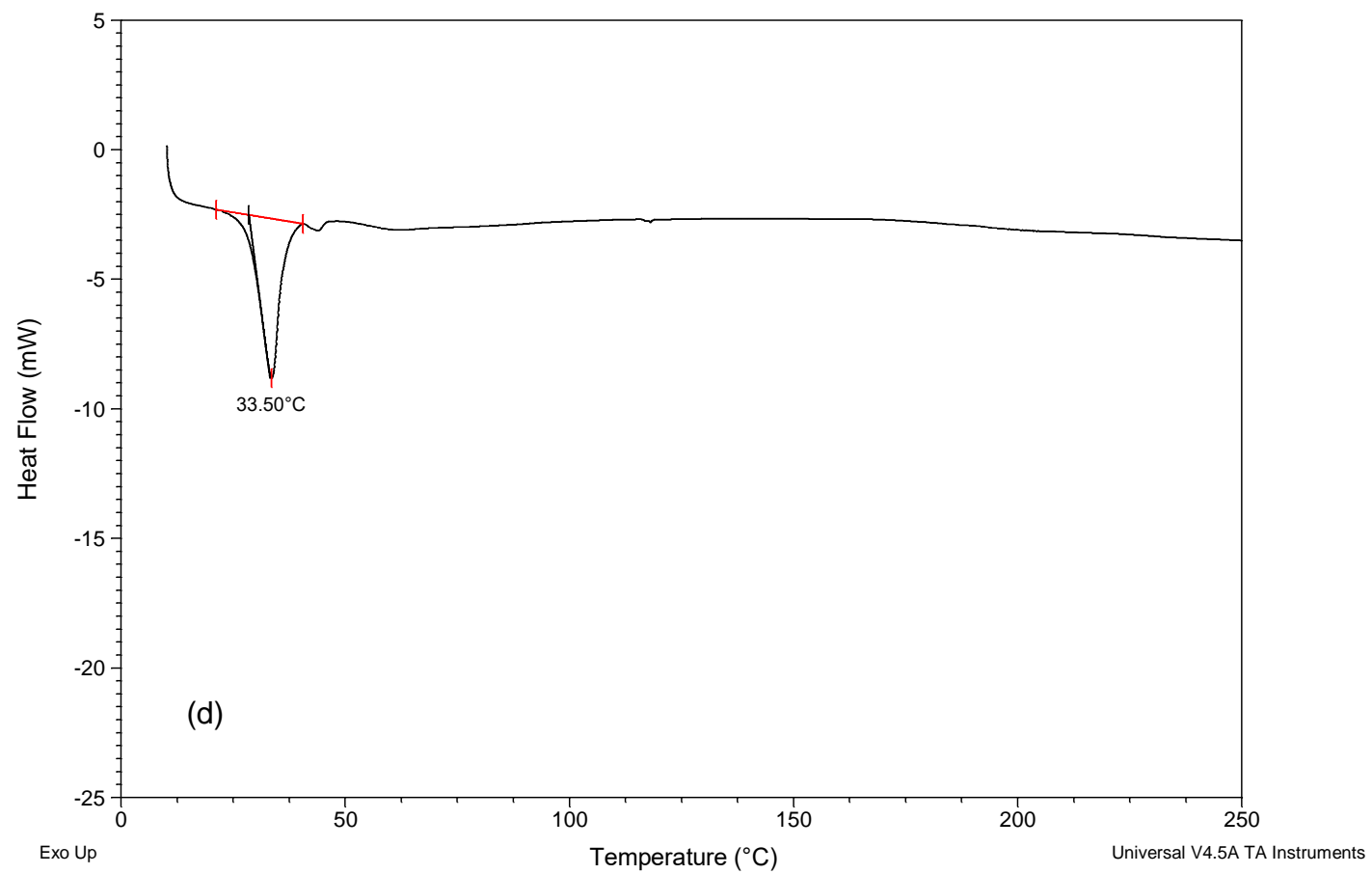

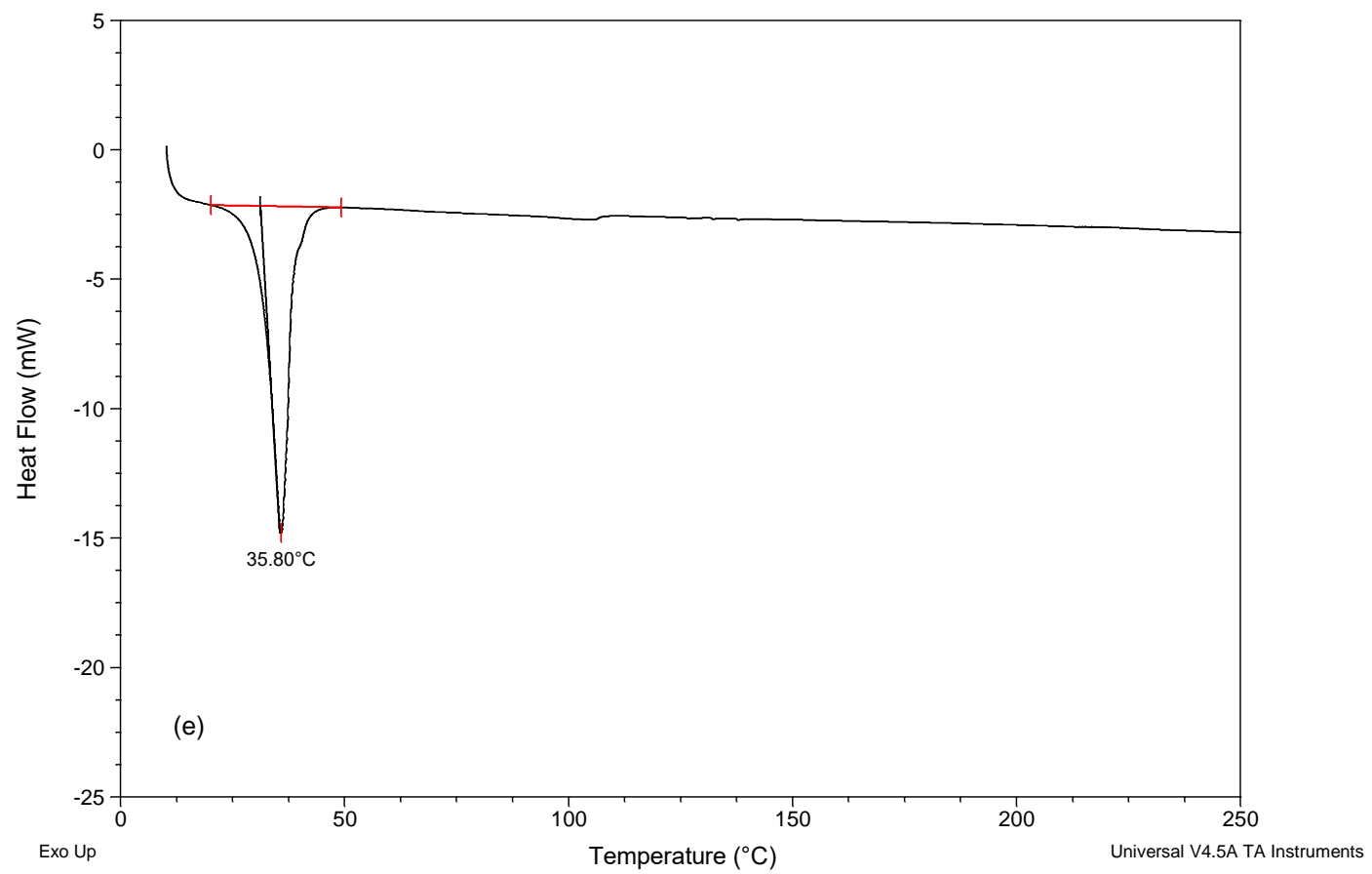

Supplement: Supplementary file 1 [file biomolecules-12-01739-s001.zip › New Figure 5.pdf]

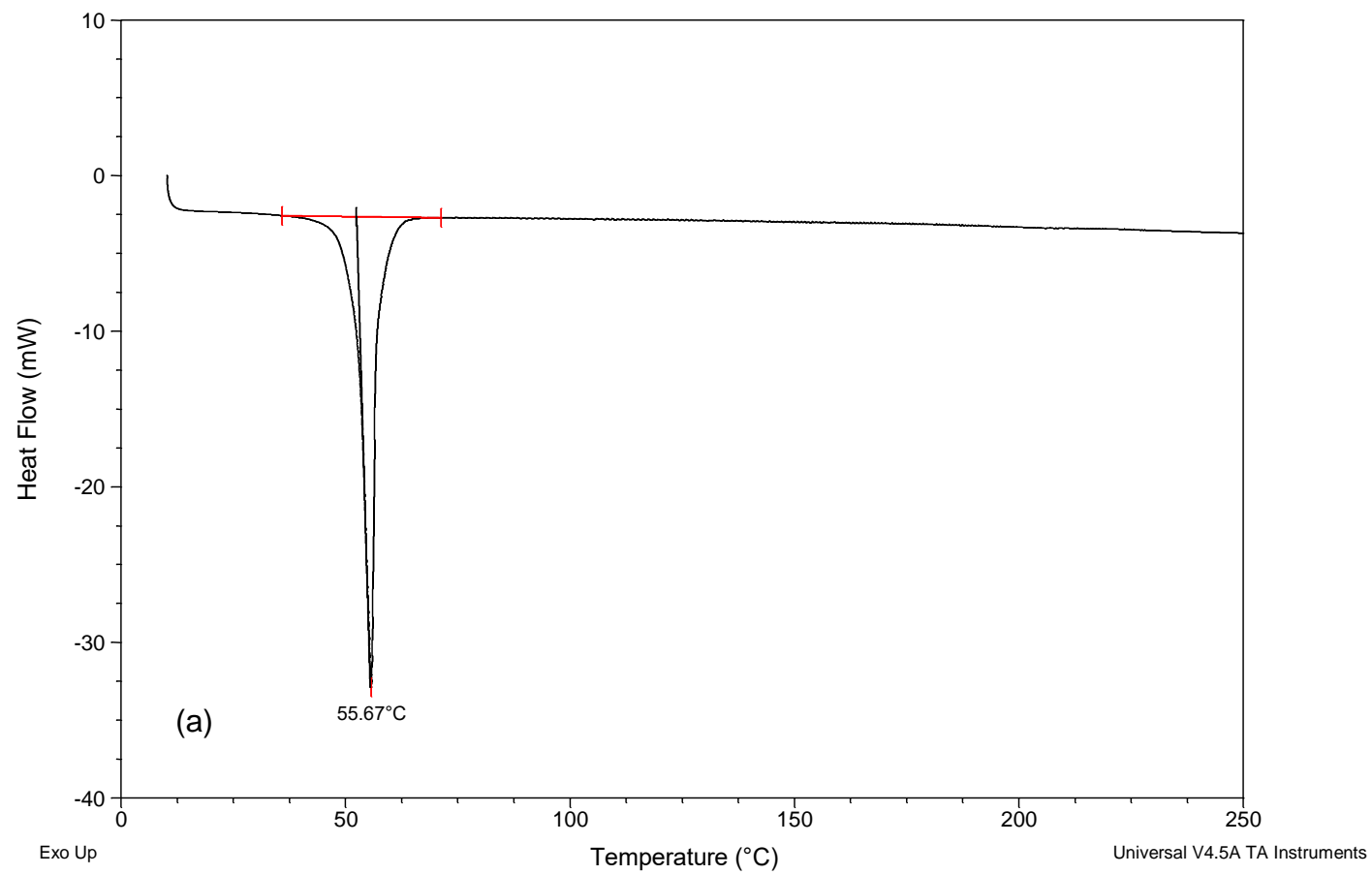

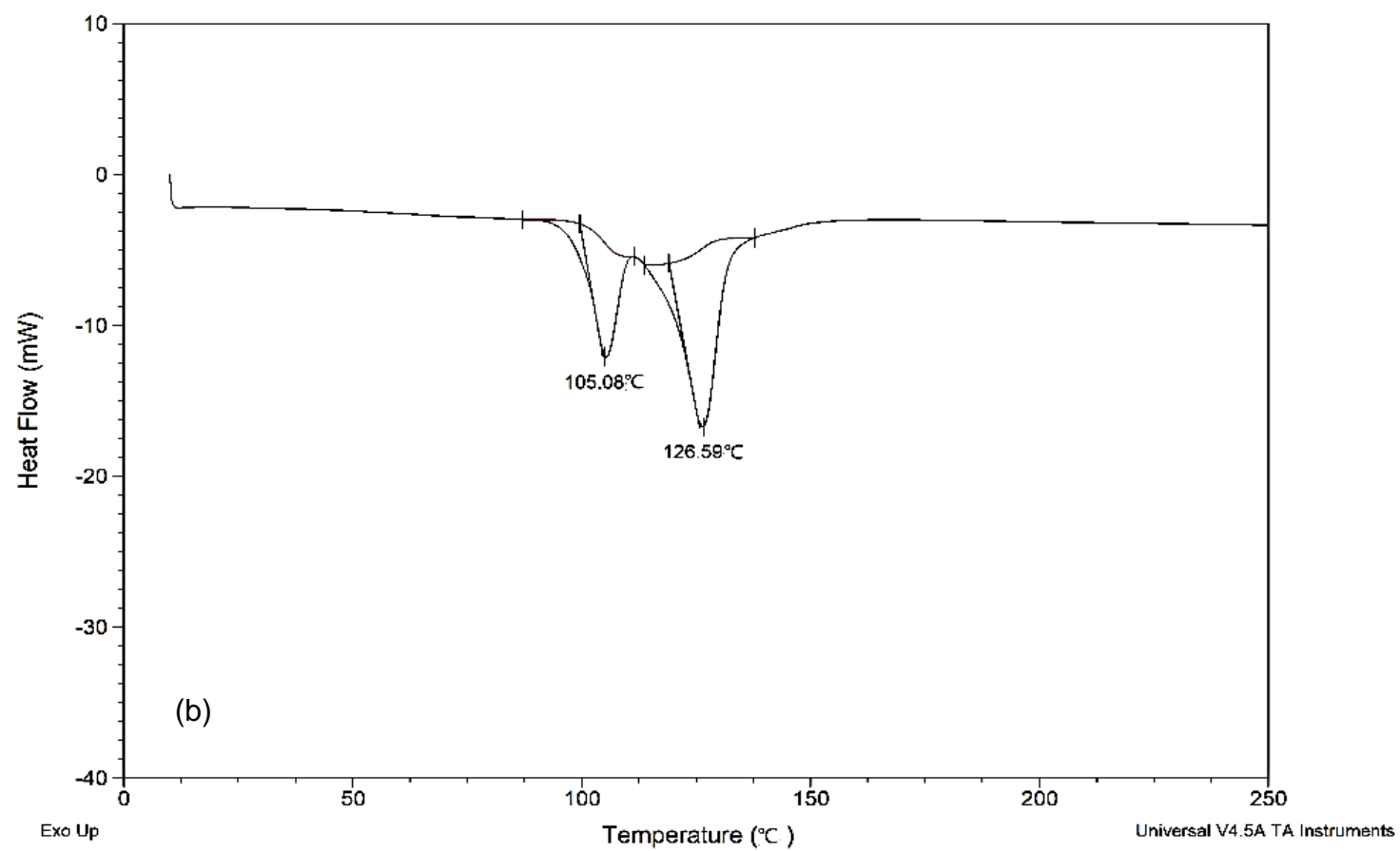

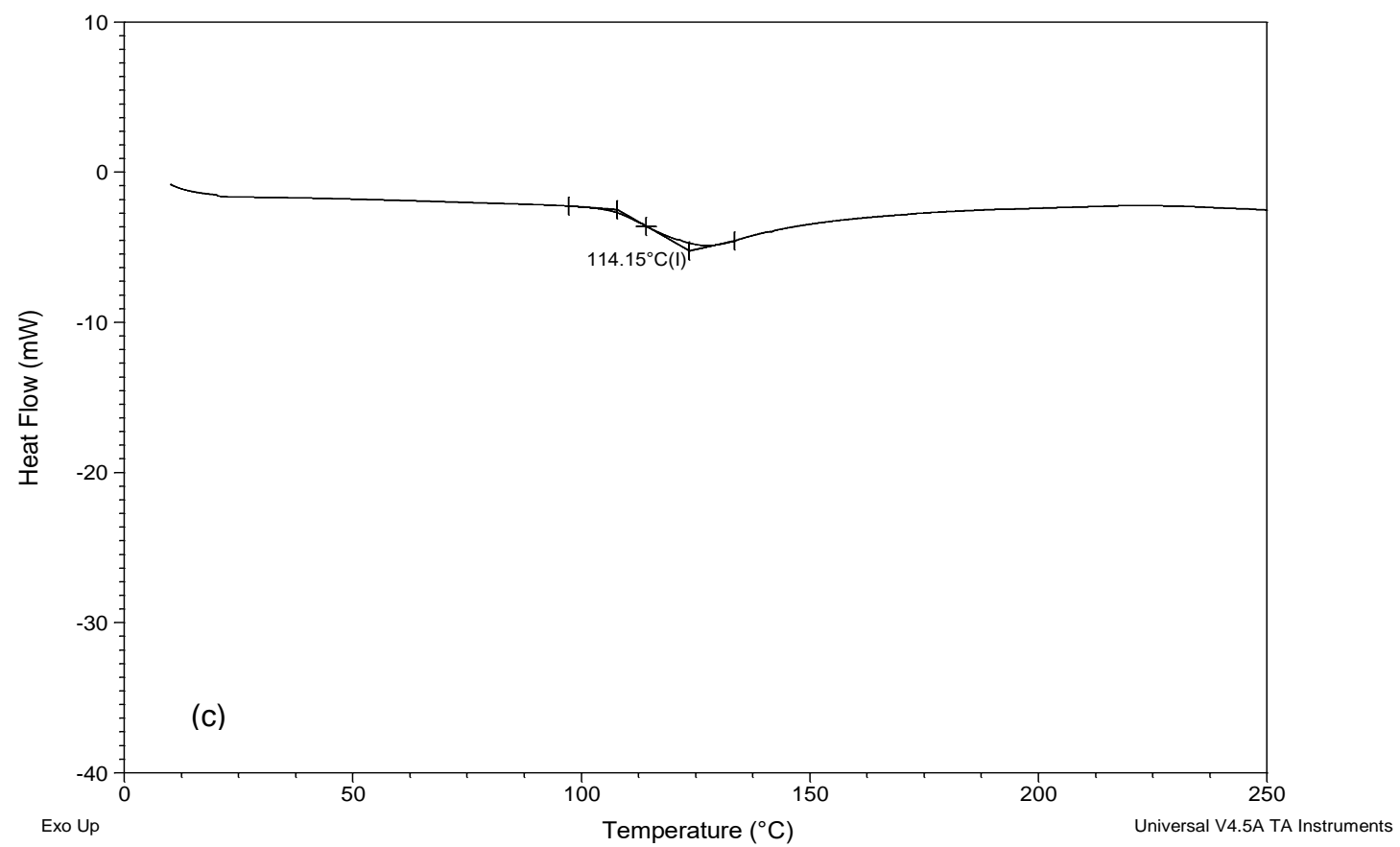

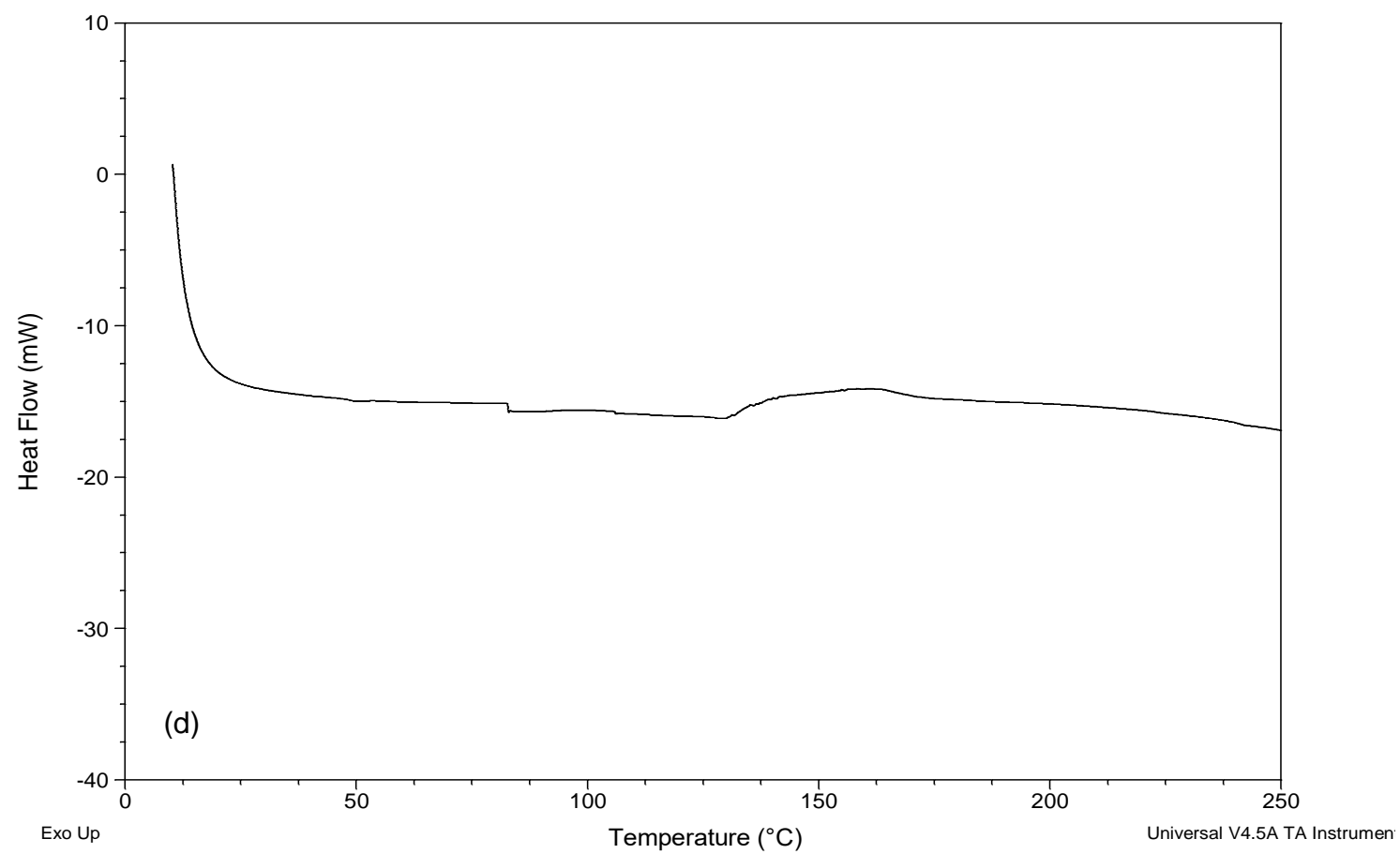

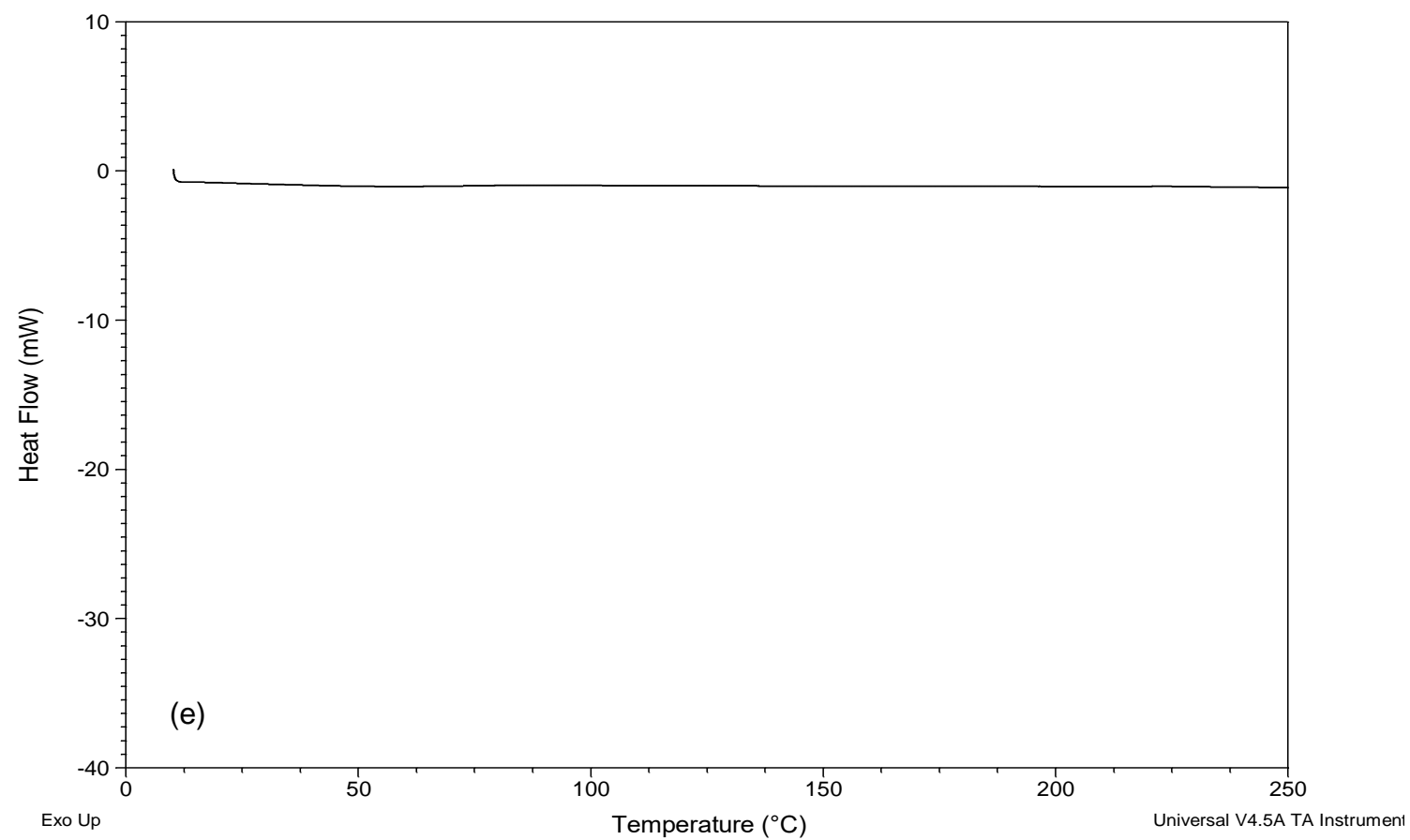

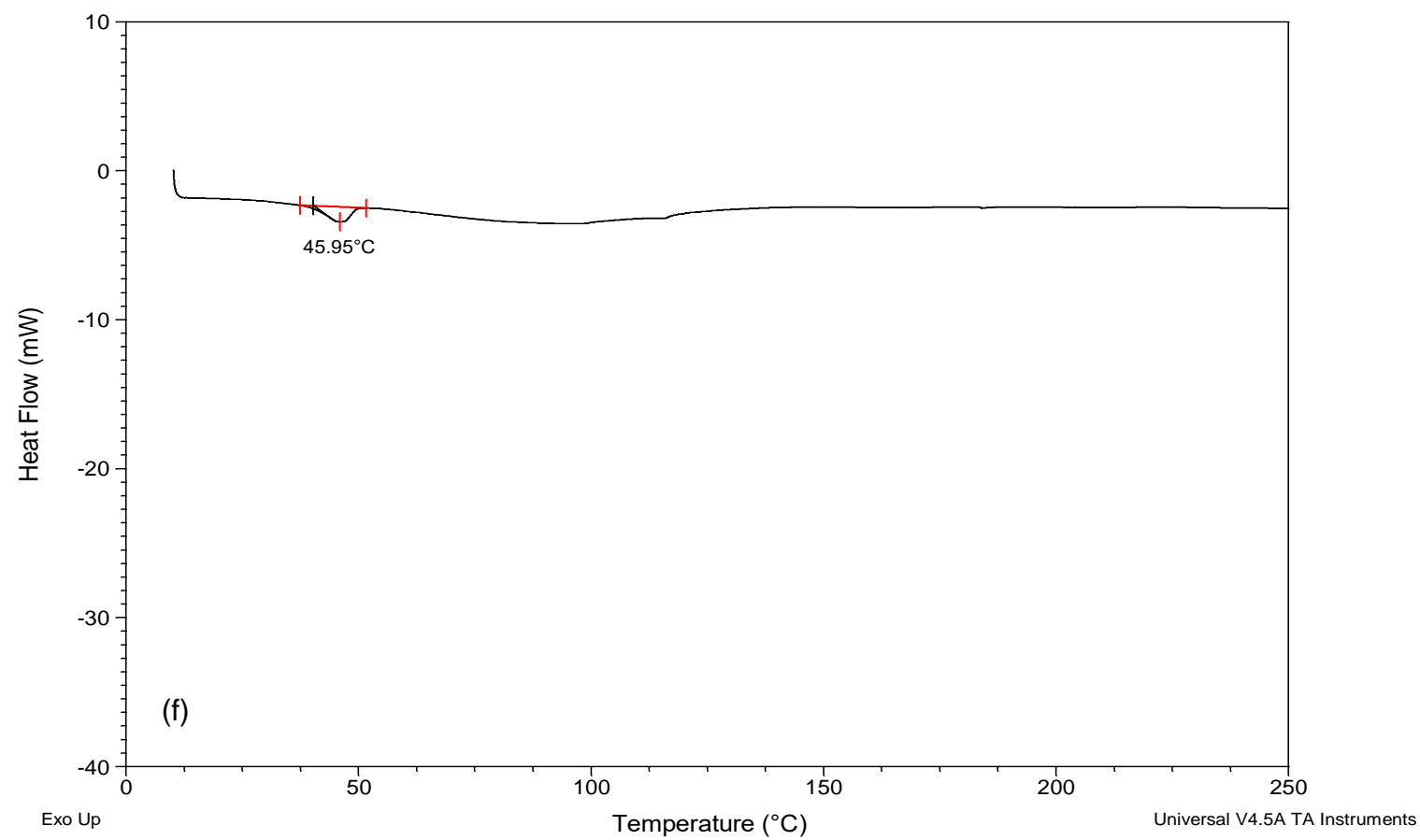

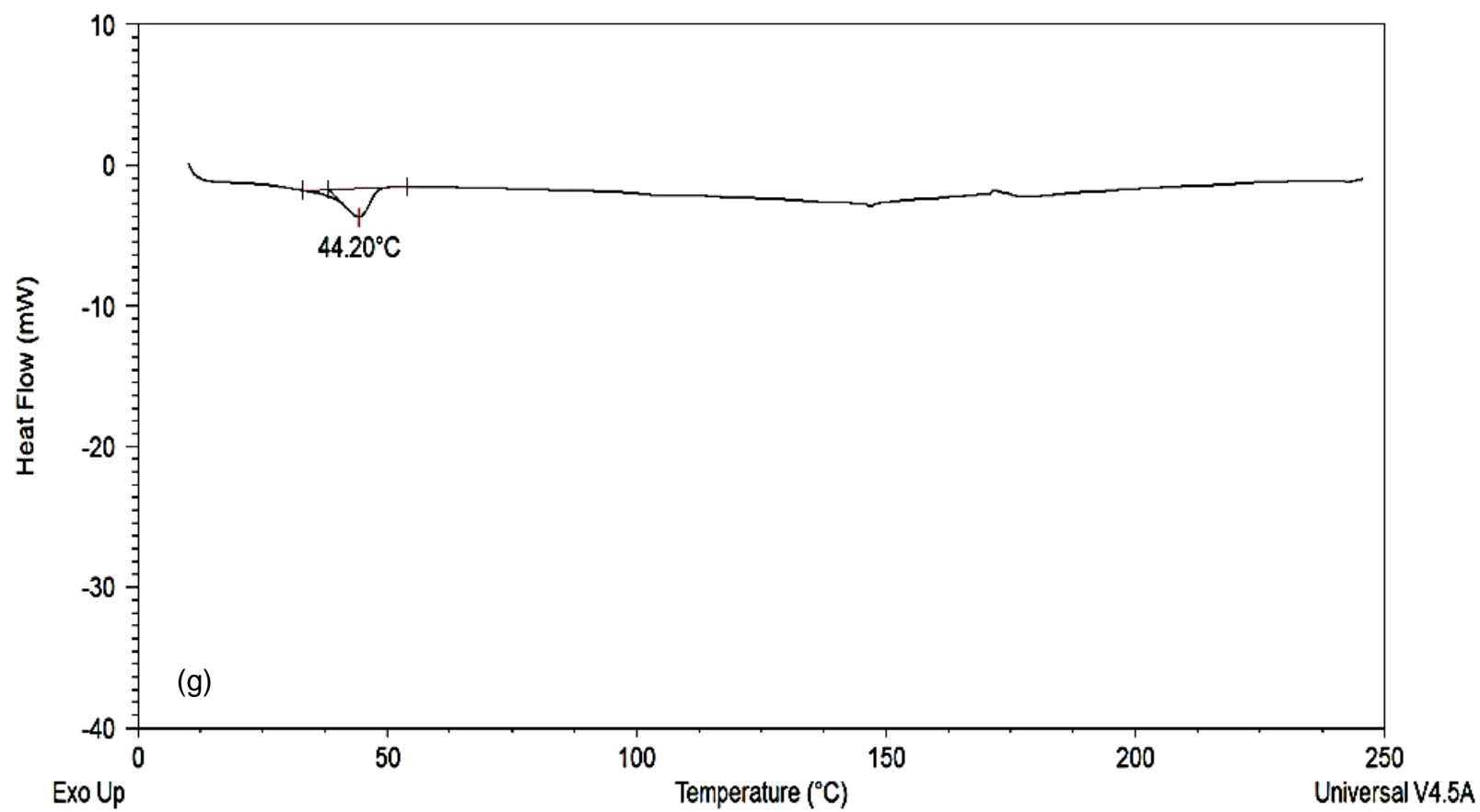

Supplement: Supplementary file 1 [file biomolecules-12-01739-s001.zip › New Figure 6.pdf]

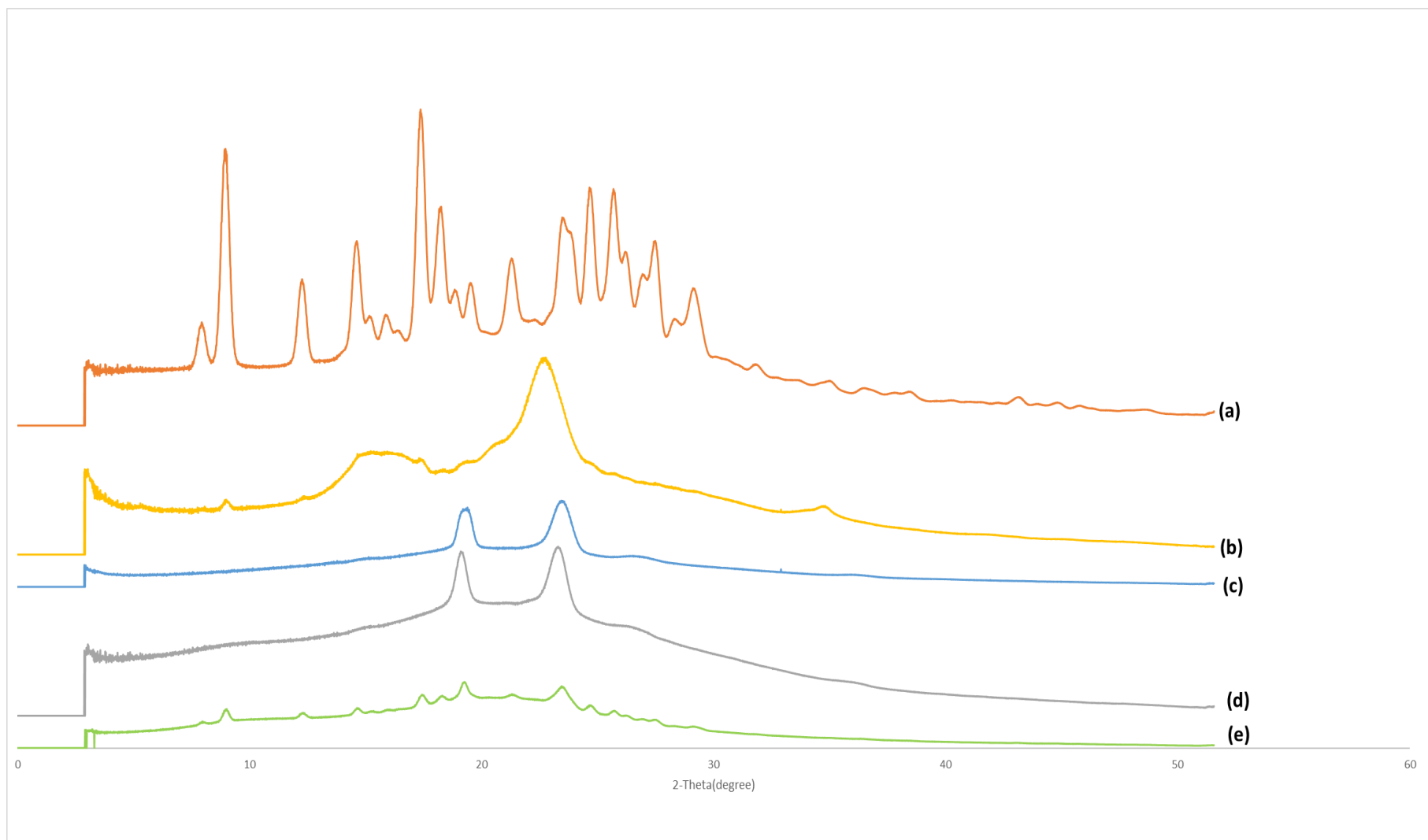

Supplement: Supplementary file 1 [file biomolecules-12-01739-s001.zip › New Figure 7.pdf]

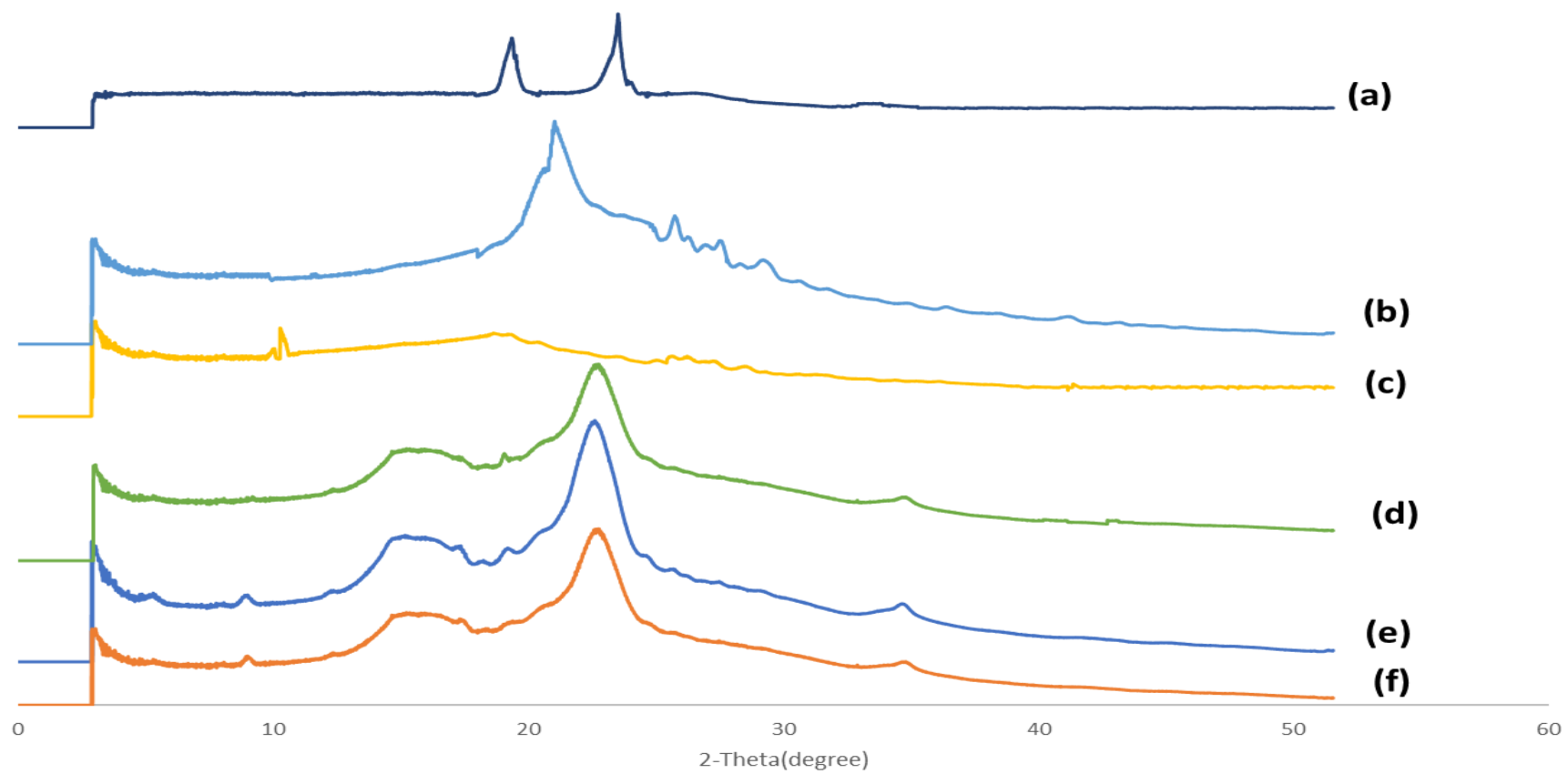

Supplement: Supplementary file 1 [file biomolecules-12-01739-s001.zip › New Figure 8.pdf]

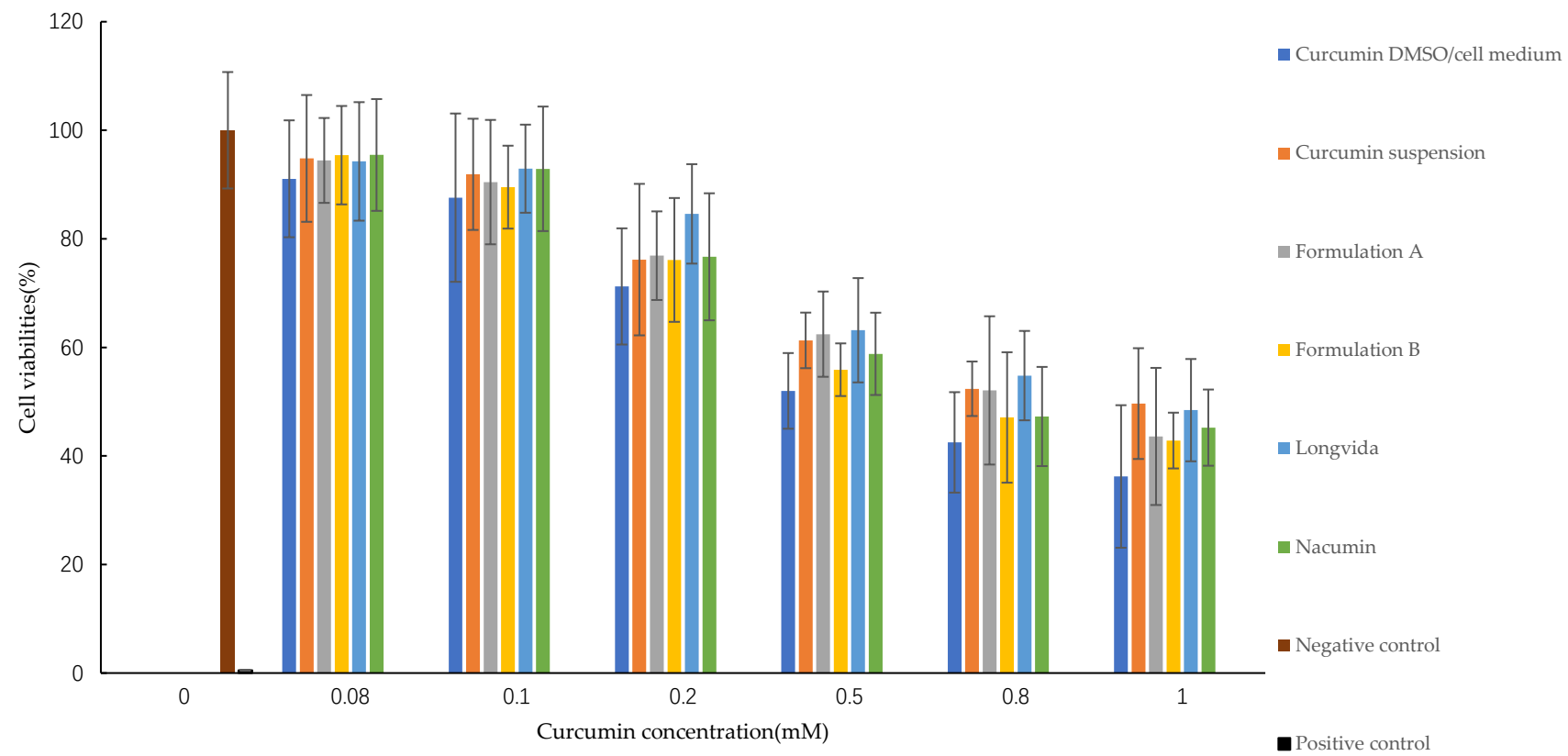

Supplement: Supplementary file 1 [file biomolecules-12-01739-s001.zip › New Figure 9.pdf]
